# High-throughput differentiation of human blood vessel organoids reveals overlapping and distinct functions of the cerebral cavernous malformation proteins

**DOI:** 10.1007/s10456-025-09985-5

**Published:** 2025-06-06

**Authors:** Dariush Skowronek, Robin A. Pilz, Valeriia V. Saenko, Lara Mellinger, Debora Singer, Silvia Ribback, Anja Weise, Kevin Claaßen, Christian Büttner, Emily M. Brockmann, Christian A. Hübner, Thiha Aung, Silke Haerteis, Sander Bekeschus, Arif B. Ekici, Ute Felbor, Matthias Rath

**Affiliations:** 1 Department of Human Genetics, University Medicine Greifswald and Interfaculty Institute of Genetics and Functional Genomics, University of Greifswald, Fleischmannstraße 43, 17475 Greifswald, Germany; 2 Department of Dermatology and Venerology, Rostock University Medical Center, Rostock, Germany; 3 Leibniz Institute for Plasma Science and Technology (INP), ZIK Plasmatis, Greifswald, Germany; 4 Institute of Pathology, University Medicine Greifswald, Greifswald, Germany; 5 Institute of Human Genetics, Jena University Hospital, Friedrich Schiller University, Jena, Germany; 6 Department of Human Medicine, MSH Medical School Hamburg, Hamburg, Germany; 7 Institute of Human Genetics, Friedrich-Alexander-University (FAU) Erlangen-Nürnberg and Universitätsklinikum Erlangen, Erlangen, Germany; 8 Institute for Molecular and Cellular Anatomy, University of Regensburg, Regensburg, Germany; 9 Faculty of Applied Healthcare Science, Deggendorf Institute of Technology, Deggendorf, Germany; 10 Institute for Molecular Medicine, MSH Medical School Hamburg, Hamburg, Germany; 11 Organoid Expertise Center, MSH Medical School Hamburg, Hamburg, Germany

**Keywords:** Blood vessel organoids, Human induced pluripotent stem cells, CRISPR/Cas9 genome editing, Cerebral cavernous malformations, Single-cell RNA sequencing

## Abstract

Cerebral cavernous malformations (CCMs) are clusters of thin-walled enlarged blood vessels in the central nervous system that are prone to recurrent hemorrhage and can occur in both sporadic and familial forms. The familial form results from loss-of-function variants in the *CCM1*, *CCM2*, or *CCM3* gene. Despite a better understanding of CCM pathogenesis in recent years, it is still unclear why *CCM3* mutations often lead to a more aggressive phenotype than *CCM1* or *CCM2* variants. By combining high-throughput differentiation of blood vessel organoids from human induced pluripotent stem cells (hiPSCs) with a *CCM1*, *CCM2*, or *CCM3* knockout, single-cell RNA sequencing, and high-content imaging, we uncovered both shared and distinct functions of the CCM proteins. While there was a significant overlap of differentially expressed genes in fibroblasts across all three knockout conditions, inactivation of *CCM1*, *CCM2*, or *CCM3* also led to specific gene expression patterns in neuronal, mesenchymal, and endothelial cell populations, respectively. Taking advantage of the different fluorescent labels of the hiPSCs, we could also visualize the abnormal expansion of *CCM1* and *CCM3* knockout cells when differentiated together with wild-type cells into mosaic blood vessel organoids. In contrast, *CCM2* knockout cells showed even reduced proliferation. These observations may help to explain the less severe clinical course in individuals with a pathogenic variant in *CCM2* and to decode the molecular and cellular heterogeneity in CCM disease. Finally, the excellent scalability of blood vessel organoid differentiation in a 96-well format further supports their use in high-throughput drug discovery and other biomedical research studies.

**Supplementary Information:**

The online version contains supplementary material available at 10.1007/s10456-025-09985-5.

## Introduction

Cerebral cavernous malformations (CCMs), sometimes also called cavernomas, cavernous angiomas, or cavernous hemangiomas, are leaky vascular lesions in the brain and spinal cord that can cause seizures, intracranial hemorrhages (ICH), or non-hemorrhagic focal neurological deficits (NH-FND) [1, 2]. With a prevalence of approximately 1 in 200, CCM is one of the most common cerebrovascular diseases. Although most CCMs are sporadic, there is also a familial form of CCM which accounts for almost 10 to 20% of all cases and differs from sporadic cases at the clinical, neuroradiological and molecular levels [1, 3–7]. Since the first description of a CCM family by H. Kufs in 1928 [8] and the identification of the three disease genes *CCM1* (also known as *KRIT1*), *CCM2*, and *CCM3* (also known as *PDCD10*) between 1999 and 2005 [9–13], CCM research has developed rapidly. Extensive studies in different cell culture models, human CCM tissue samples, and mouse, zebrafish, and other model organisms have contributed significantly to our current understanding of CCM pathogenesis [14–16]. It is now well established that although familial CCM is a classic autosomal dominant disorder, the inactivation of *CCM1*, *CCM2,* or *CCM3* is recessive at the cellular level [15]. The deregulation of key signaling pathways that initiate CCM formation, e.g., gain of MEKK3-KLF2/4 signaling or increase of RhoA/ROCK activity [17, 18], is only triggered by the biallelic inactivation of a *CCM* gene. Furthermore, next-generation sequencing of bulk and single nucleus DNA from human CCMs has shown that the proportion of cells with a biallelic *CCM* gene variant is often rather low within the vascular lesions [19–21]. While in vivo and in vitro research has also led to the first phase 1 and 2 clinical trials, no pharmacological CCM therapy has yet been approved for clinical use [22–24]. Thus, neurosurgical treatment often remains the only option for symptomatic CCMs, especially after a previous hemorrhage or when they cause medically refractory seizures [1]. Since surgical resection is always associated with the risk of postoperative morbidity, the development of new CCM drugs remains a top priority in research. The increasing availability of advanced in vitro methods is raising hopes in many fields of biomedical sciences to significantly accelerate drug discovery. Organoid cultures, one of these advanced in vitro methods, have already enabled a wide range of new applications in basic and translational research, regenerative medicine, and drug discovery [25]. Using specific growth factors, small molecules, extracellular matrix components, and cell culture conditions, human pluripotent stem cells can be very effectively differentiated into brain, lung, kidney, liver, and other specific organoid types [26]. For example, the differentiation of human blood vessel organoids, first described by Wimmer and colleagues [27, 28], now provides new insights into the complex processes of vasculogenesis and angiogenesis in health and disease. Especially in combination with CRISPR/Cas9 genome editing in fluorescently labeled human induced pluripotent stem cells (hiPSCs), human blood vessel organoids can help to better understand how wild-type (WT) and knockout (KO) cells interact within the CCM lesions. This aspect of CCM pathogenesis is still poorly understood but could represent a promising target to suppress CCM development or progression [29]. Using mosaic KO/WT blood vessel organoids differentiated from mEGFP-tagged *CCM3* KO and mTagRFPT-tagged *CCM3* WT hiPSCs, we recently directly visualized a significantly increased KO cell proliferation [30]. This abnormal in vitro behavior perfectly reflects the in vivo situation, where a clonal dominance of *Ccm3* KO endothelial cells was observed in CCM mouse models with a Confetti fluorescence reporter system [31, 32]. Evidence of abnormally increased proliferation has also been observed in *CCM1* and *CCM2* KO cells [33–36]. However, the literature on *CCM2* is still inconsistent [37, 38].

Despite their many advantages, vascular organoid cultures have been challenging to scale up, e.g., for high-throughput studies. To address this limitation, we have established a protocol that eliminates the most labor-intensive step of manually extracting vascular networks from the extracellular matrix, allowing the differentiation of human blood vessel organoids in a 96-well format. Furthermore, the protocol does not require animal collagen I and bovine calf serum. Using this high-throughput compatible and nearly xeno-free protocol in combination with single-cell RNA sequencing (scRNA-seq) analysis, we demonstrate that blood vessel organoids are powerful tools for CCM research. Compared to the C. elegans, zebrafish, and mouse models [39] as well as the recently described sophisticated pluripotent stem cell-based blood–brain barrier assembloid model [40], our blood vessel organoid model is characterized by its excellent scalability. This makes it possible to gain deeper insight into the cellular and molecular consequences of *CCM1*, *CCM2,* or *CCM3* inactivation within a relatively short time and with reasonable effort. Its scalability is also an advantage for high-throughput screening approaches.

## Materials and methods

### Cell culture

Human induced pluripotent stem cells (hiPSCs) were cultured in 6-well plates (Greiner Bio-One, Frickenhausen, Germany, #657160) using Essential 8 Flex medium (Thermo Fisher Scientific, Waltham, MA, USA, #A2858501). Cell culture dishes were pre-coated with growth factor-reduced Matrigel (Corning Inc., Corning, NY, USA, #356231). HiPSCs were thawed and plated at a density of 10,000 cells/cm^2^ in Matrigel-coated 6-well plates in the presence of RevitaCell supplement (1:100) (Thermo Fisher Scientific, #A2644501). HiPSC cultures were regularly tested negative for mycoplasma contamination by PCR. The hiPSC lines AICS-0036–006 [WTC-mEGFP-Safe harbor locus (AAVS1)-cl6 (mono-allelic tag); hPSCreg ID: UCSFi001-A-12; RRID:CVCL_JM19], AICS-0054–091 [WTC-mTagRFPT-CAAX-Safe harbor locus (AAVS1)-cl91 (mono-allelic tag); hPSCreg ID: UCSFi001-A-23; RRID:CVCL_VK84] and AICS-0016–184 [WTC-mEGFP-ACTB-cl184 (mono-allelic tag); hPSCreg ID: UCSFi001-A-3; RRID:CVCL_JM16], which are part of the Allen Cell Collection (Allen Institute for Cell Science, Seattle, WA, USA), were purchased from the Coriell Institute (Camden, NJ, USA). *CCM1* and *CCM2* knockout (KO) AICS-0036-006 hiPSCs as well as *CCM1, CCM2,* and *CCM3* KO AICS-0016-184 hiPSCs were generated in this study following our established protocols [30, 41]. The generation of *CCM3* KO AICS-0036-006 hiPSCs has been described earlier [30]. "*CCM1* KO", "*CCM2* KO", and "*CCM3* KO" hereafter refer to KO cells derived from AICS-0036-006-hiPSCs unless otherwise specified. For CRISPR/Cas9 genome editing, single guide RNA (sgRNA):Cas9 ribonucleoprotein complexes with the following target sequences were used: *CCM1* 5′-GGAGCTCCTAGACCAAAGTA-3′; *CCM2* 5′-GGTCAGTTAACGTCCATACC-3′; *CCM3* 5′-CAACTCACCTCATTAAACAC-3′ (Integrated DNA Technologies, Coralville, IA, USA). The introduction of homozygous or compound heterozygous loss-of-function variants by CRISPR/Cas9 editing in the generated clones was verified by amplicon-based next-generation sequencing. HiPSC quality control was performed by karyotyping and immunofluorescence analysis of the pluripotency markers OCT4, SSEA4, SOX2, and TRA-1-60 using the Pluripotent Stem Cell 4-Marker Immunocytochemistry Kit (Thermo Fisher Scientific, #A24881) as previously described [41].

### Generation of hiPSC, mesodermal, and vascular aggregates

For the generation of hiPSC aggregates and their differentiation into vascular aggregates, the previously described protocol of Wimmer and colleagues [27, 28] was modified to allow aggregate formation in a 96-well format. Briefly, on day -2, hiPSCs were detached and dissociated using StemPro Accutase (Thermo Fisher Scientific, #A1110501). Cells were counted and diluted to a concentration of 1,000 cells/well in fresh aggregation medium [27, 28] containing 50 μM Y-27632 (Stemcell Technologies, Vancouver, Canada, #72304). Cells were seeded in 100 μL medium per well in ultra-low attachment PrimeSurface 96 Slit-well plates (Sbio, Hudson, NH, USA, #MS9096SZ). Plates were centrifuged at 300 × g for 3 min at room temperature and incubated at 37 °C and 5% CO_2_ for 48 h to promote effective hiPSC aggregation. On day 0, the PrimeSurface 96 Slit-well plates were washed by adding 25 mL of N2B27 medium to the plate using a 25 mL serological pipette. The plate was gently tilted to all sides to distribute the medium evenly. The medium was removed by tilting the 96 Slit-well plate and slowly aspirating it from one corner of the plate with a 25 mL serological pipette. Next, 25 mL of N2B27 medium + 12 µM CHIR99021 (Tocris Bioscience, Bristol, United Kingdom, #4423) + 30 ng/mL BMP-4 (Miltenyi Biotec, Bergisch Gladbach, Germany, #130-111-164) were added. The cells were incubated at 37 °C and 5% CO_2_ for 72 h to induce mesodermal differentiation. On day 3, 25 mL of the old medium were aspirated, the aggregates were washed as described above, and 25 mL of N2B27 medium + 100 ng/mL VEGF-A (Peprotech, Hamburg, Germany, #100–20-50) + 2 µM forskolin (Miltenyi Biotec, #130-117-341) were added. The cells were incubated at 37 °C and 5% CO_2_ for 48 h to induce vascular differentiation. To study aggregation efficiency, KO and WT hiPSCs were seeded with different cell densities ranging from 200 cells/well to 1,800 cells/well. Following the protocol of mesodermal and endothelial differentiation described above, the aggregates were imaged on days 0, 3, and 5 (= hiPSC, mesodermal, and vascular aggregates, respectively). The imaging was performed using an EVOS M5000 imaging systems microscope (Thermo Fisher Scientific). The size of the aggregates was determined using FIJI v.1.54 [42]. The aggregates were fixed with 4% PFA on day 5. The following primary and secondary antibodies were used for immunofluorescent evaluation: ZO-1 (Novus Biologicals, Centennial, CO, USA, 1:100, #NBP1-85047), anti-rabbit IgG Alexa 647 (Thermo Fisher Scientific, 1:200 #A21246).

### Generation of human blood vessel organoids

For the generation of vascular networks and blood vessel organoids, the previously described protocol by Wimmer and colleagues [27, 28] was modified as follows: On day 5, vascular aggregates were transferred to a 96-well Akura plate (InSphero, Schlieren, Switzerland, #CS0900403) and embedded in a human collagen I/Matrigel matrix. The following amounts of human collagen I (Advanced BioMatrix, Carlsbad, CA, USA, #5007-20ML) and Matrigel solution were used for one 96-well Akura plate: 490 µL each for the first and second matrix layers (367.5 µL of human collagen I solution + 122.5 µL of Matrigel). Matrigel and human collagen I were kept on ice. To form the first matrix layer, 3 µL of human collagen I/Matrigel solution was added to each well of an empty Akura 96-well plate. The plate was incubated at 37 °C for 2 h to solidify the matrix, while a second Akura plate was prepared as a transfer plate. To avoid air bubbles at the bottom of the transfer plate, the wells were washed with StemPro-34 serum-free medium (StemPro-34 SFM, Thermo Fisher Scientific, #10,639,011). After removing 10 mL of the N2B27 medium from the PrimeSurface 96 Slit-well plate containing the vascular aggregates, a multichannel pipette with cut pipette tips was used to aspirate the vascular aggregates in 70 µL medium from the bottom of the Slit-well plate. They were transferred to the transfer plate, and excess medium was removed. 50 µL of StemPro-34 SFM was then added to each well before 40 µL of medium containing the aggregates was transferred to the Akura plate containing the first collagen I/Matrigel matrix layer using a multichannel pipette with cut pipette tips. Once again, as much medium as possible was aspirated before 5 µL of human collagen I/Matrigel solution was slowly added as a second matrix layer embedding the aggregates. For solidification of the matrix, the plate was incubated for 2 h at 37 °C. Meanwhile, StemPro-34 SFM containing 15% Panexin CD (PAN Biotech, Aidenbach, Germany, #P04-930500) was prepared in a Falcon tube and heated to 37 °C in a water bath for 30 min. 80 µL of StemPro-34 SFM supplemented with 15% Panexin CD, 100 ng/mL FGF-2 (Miltenyi Biotec, #130-093-564), and 100 ng/mL VEGF-A were carefully added to the center of each well. The plate was incubated at 37 °C and 5% CO_2_ to induce sprouting. The medium was replaced on days 7 and 10. On day 12, the matrix plugs with the vascular networks were transferred without manual extraction from the gel to individual wells of a PrimeSurface 96 Slit-well plate by pipetting. Medium was changed as previously described by adding 25 mL of StemPro-34 SFM supplemented with 15% Panexin CD, 100 ng/mL FGF-2, and 100 ng/mL VEGF-A to the Slit-well plate. Over the next two days, the networks were regularly pipetted up and down with cut pipette tips to promote the dissolution of excess matrix. The medium was exchanged again on day 14. On day 17, the blood vessel organoids were ready for following analyses. To generate mosaic blood vessel organoids, mTagRFPT-tagged AICS-0054 WT hiPSCs and *CCM1* KO, *CCM2* KO, or *CCM3* KO mEGFP-tagged AICS-0036 hiPSCS were mixed in a 19:1 ratio and differentiated to blood vessel organoids as described above. For immunofluorescence analysis, vascular networks and blood vessel organoids were fixed in 1% PFA for 30 min or 1 h, respectively. Antibody staining was performed as described elsewhere [27]. The following primary and secondary antibodies were used: CD31 (RnD Systems, Minneapolis, MN, USA, #BBA7, 1:50), PDGFR-β (Cell Signaling Technology, Danvers, MA, USA, #3169S, 1:50), VE-cadherin (Cell Signaling Technology, #2500S, 1:400), ZO-1 (Novus Biologicals, 1:100, #NBP1-85-047), anti-mouse IgG Fluor 350 (Thermo Fisher Scientific, #A-11045), anti-rabbit IgG Alexa Fluor 647 (Thermo Fisher Scientific, 1:200, #A21246), anti-mouse IgG Alexa 555 (Abcam, Cambridge, England, 1:200, #ab150114). Nuclei were stained with Hoechst 33342. Imaging was performed using an Operetta CLS imaging system (PerkinElmer, Waltham, MA, USA) in non-confocal mode or a Stellaris 8 (Leica, Wetzlar, Germany) microscope.

### Perfusion of human blood vessel organoids in organoplates graft plates

In vitro perfusion assays were performed with OrganoPlate Graft plates (Mimetas, Oegstgeest, Netherlands, #6401-400-B). Gel preparation was performed according to the manufacturer's instructions. 3 µL of gel containing 4 mg/mL rat tail Collagen I (Ibidi, Gräfelfing, Germany, #50201), 100 mM HEPES (Thermo Fisher Scientific, #15630106), and 3.7 mg/mL NaHCO_3_ (Merck, Darmstadt, Germany, #6329) were added to the gel inlet of the OrganoPlate. For polymerization of the matrix, the plate was incubated for 15 min at 37 °C and 5% CO_2_. Human umbilical vein endothelial cells (HUVECs, PromoCell, Heidelberg, Germany) were cultured in endothelial cell growth medium (ECGM, PromoCell) supplemented with 10% fetal bovine serum (FBS) (Thermo Fisher Scientific, #A5670701). Following the manufacturer's instructions, 2 μL of 1 × 10^4^ cells/μL were seeded into the perfusion inlets of the Organoplate. After adding 50 µL of medium to the perfusion inlets, the plate was incubated for 2–3 h at 37 °C and 5% CO_2_. After cell attachment, 50 µL of medium was added to the perfusion outlets, and the plate was placed on an interval MIMETAS rocker with an inclination of 14° and an interval of 8 min. Medium was replaced every two to three days. Cells were cultured for three days until tube formation was completed. To initiate sprouting, 50 µL of medium mixed with an angiogenic cocktail of 37.5 ng/mL VEGF-A, 37.5 ng/mL FGF-2, 37.5 ng/mL rhMCP-1 (ImmunoTools, Friesoythe, Germany, #11343384), 37.5 ng/mL rhHGF (ImmunoTools, #11343413), 250 nM S1P (Sigma Aldrich, St. Louis, MO, USA, #S9666) and 37.5 ng/mL PMA (Sigma Aldrich, # P1585) were added to the graft chamber according to the manufacturer's instruction. To graft hiPSC-derived vascular networks in the OrganoPlates, medium was removed from all channels after six days of sprouting. Next, perfusion inlets and outlets were filled with ECGM supplemented with 10% FBS. StemPro-34 SFM containing 15% PCD and the previously described angiogenic cocktail was added to the graft chamber. Finally, the vascular networks were transferred into the graft chamber using cut pipette tips. Medium was replaced every two to three days. After five days, the perfusion of the vascular network was visualized by adding medium containing 0.5 mg/mL 150 kDa tetramethylrhodamine (TMR)-amino dextran (Fina Biosolutions LLC, Rockville, MD, USA, #TMR-Amdex150K) to the left perfusion inlet and outlet. After 15 min, the plate was fixed using 4% PFA for 30 min and imaged according to the manufacturer's instructions.

### Grafting of organoids on chicken chorioallantoic membranes (CAM model)

To study perfusion of the organoids in the chorioallantoic membrane (CAM) 3D in vivo model, complete blood vessel organoids were resuspended in StemPro-34 SFM medium containing 15% PCD and 10% DMSO, frozen in liquid nitrogen, and shipped overnight. Fertilized chicken eggs were rinsed with water, brushed, and disinfected with 70% ethanol. Afterward, they were immediately placed on a rotation device inside a ProCon egg incubator (Grumbach, Asslar, Germany) at a constant temperature of 37.8 °C, a pCO_2_ of 5%, and with the humidity calibrated to 63%. On the fourth day of incubation, two windows were cut into the eggshell [43–45]. The first window, that was located at the air entrapment of the egg, enabled an equilibrium of pressure inside the egg and was permanently closed with Leukosilk (BSN Medical, Hamburg, Germany). The second window was cut into the longitudinal part of the egg and covered with a removable strip of Leukosilk. After organoid engraftment, the eggs were placed on glass trays containing autoclaved sand in the non-rotating compartment of the egg incubator. After a one-week growth period of the organoids on the CAM, they were explanted and prepared for histological analysis. First, the organoids were immersion fixed in 4% paraformaldehyde (PFA) under constant movement for 7 days at 4 °C. After removing the PFA, the organoids were washed in PBS (phosphate buffered saline, pH 7.4) for 30 min under constant movement. This procedure was repeated three times. Consequently, the samples were dehydrated and embedded in paraffine. Tissue samples of a thickness of 6 µm were cut using a microtome and stained with hematoxylin and eosin (H&E staining).

### Single-cell RNA sequencing (scRNA-seq)

After blood vessel organoid synthesis, ten organoids per genotype were pooled, and enzymatic dissociation was performed. First, blood vessel organoids were transferred to a sterile Petri dish and minced with a scalpel. Second, the minced organoids were then suspended in 1 mL enzymatic mix containing 4 mg Liberase TH solution (Sigma Aldrich), 30 mg Dispase II (Sigma Aldrich), and 0.5 mg DNase I solution (New England Biolabs, Ipswich MA, USA) in PBS and incubated at 37 °C and 5% CO_2_ for 30 min. Third, the dissociated blood vessel organoids were mixed and passed through a 70 µm cell strainer. 3 mL of DMEM/F-12 (Thermo Fisher Scientific, #11330032) with 10% FBS (Thermo Fisher Scientific, #A5670701) was added to stop the enzymatic reaction. Next, cells were centrifuged at 300 × g for 5 min, resuspended in StemPro-34 medium with 10% DMSO, and frozen in liquid nitrogen for shipment. Library preparation for scRNA-seq was performed with the Chromium Single Cell Next GEM 3' Reagent Kit v3.1 (10 × Genomics, Pleasanton, CA, USA) on a Single Cell 3' device following manufacturer's instructions. Sequencing was performed on a Novaseq 6000 sequencer (Illumina, San Diego, CA, USA). Cellranger version 7.0.1 was used to generate read data, demultiplex single cells, perform sequence alignment, and generate raw count data. Further filtering of raw data, generation of clusters, and analysis of gene expression differences were performed with Seurat 4.0.4. In brief, cells with a UMI count under 2,500 and less than 600 detected genes were removed, and the mitochondrial DNA and ribosomal RNA cut-off values were set to 10% and 5%, respectively. Seurat SCTransform was further used to normalize samples. Data was integrated using a mutual nearest neighbors (MNN) approach and unsupervised cell clustering was performed with the Leiden algorithm. Web-based Cell-type-Specific Enrichment Analysis of Genes (WebCSEA) was applied to identify enriched cell types of generated clusters [46] using the protein-coding marker genes with a log_2_FC ≥ 0.5 for each cluster. For each cluster, The CZ CELLxGENE Discover browser [47] was used to visualize the tissue and cell type-specific expression levels of the top 10 marker genes in specific clusters. Differentially expressed genes (DEG) of individual clusters were analyzed with the ShinyGO tool [48] and subjected to gene set enrichment analyses with the GO cellular process and molecular function gene sets.

### Differentiation of hiPSCs into ECs and endothelial co-culture

*CCM1* KO AICS-0016 hiPSCs, *CCM3* KO AICS-0016 hiPSCs, WT AICS-0016 hiPSCs, and WT AICS-0054 hiPSCs were differentiated into ECs (iECs) using the STEMdiff Endothelial Differentiation Kit (Stemcell Technologies) as previously described [41]. To confirm endothelial differentiation, cells were fixed with 4% PFA at passage 1 and stained for the endothelial markers CD31 (Cell Signaling, 3528S, 1:800), VE-cadherin (2500S, 1:400, Cell Signaling) and VWF (Thermo Fisher Scientific, MA5-14029, 1:66). For endothelial co-culture assays, AICS-0016 KO iECs were mixed with AICS-0054 WT iECs in a 1:9 ratio and cultured together for 6 days in EndoGRO-MV media or STEMdiff Endothelial Expansion media on 96-well plates. Co-cultures of AICS-0016 WT iECs and AICS-0054 WT iECs were used as controls. Wells were fixed with 4% PFA, and nuclei were stained with Hoechst 33342 (Thermo Fisher Scientific, #62249). FIJI was used to compare the area of mEGFP-tagged cells with the total area.

### Statistical analysis

Statistical analysis was performed in GraphPad Prism 8 (GraphPad Software, USA). Normality was assessed using the Shapiro–Wilk test. For normally distributed data, statistical significance was evaluated using multiple two-sample t-tests with Welch's correction to account for unequal variances between groups. For non-normally distributed data, pairwise Mann–Whitney U-tests were performed, as described in the figure legends. Holm-Šídák adjustment for multiple testing was applied within each outcome category, with the following notation: ns = *P* ≥ 0.05; * = *P* < 0.05; ** = *P* < 0.01; *** = *P* < 0.001. The Pearson correlation coefficient for the co-localisation of endothelial cells and pericytes was calculated with the JaCoP plugin [49] and FIJI v.1.54.

## Results

### High-throughput-compatible and nearly xeno-free synthesis of human blood vessel organoids

**Fig. 1:**
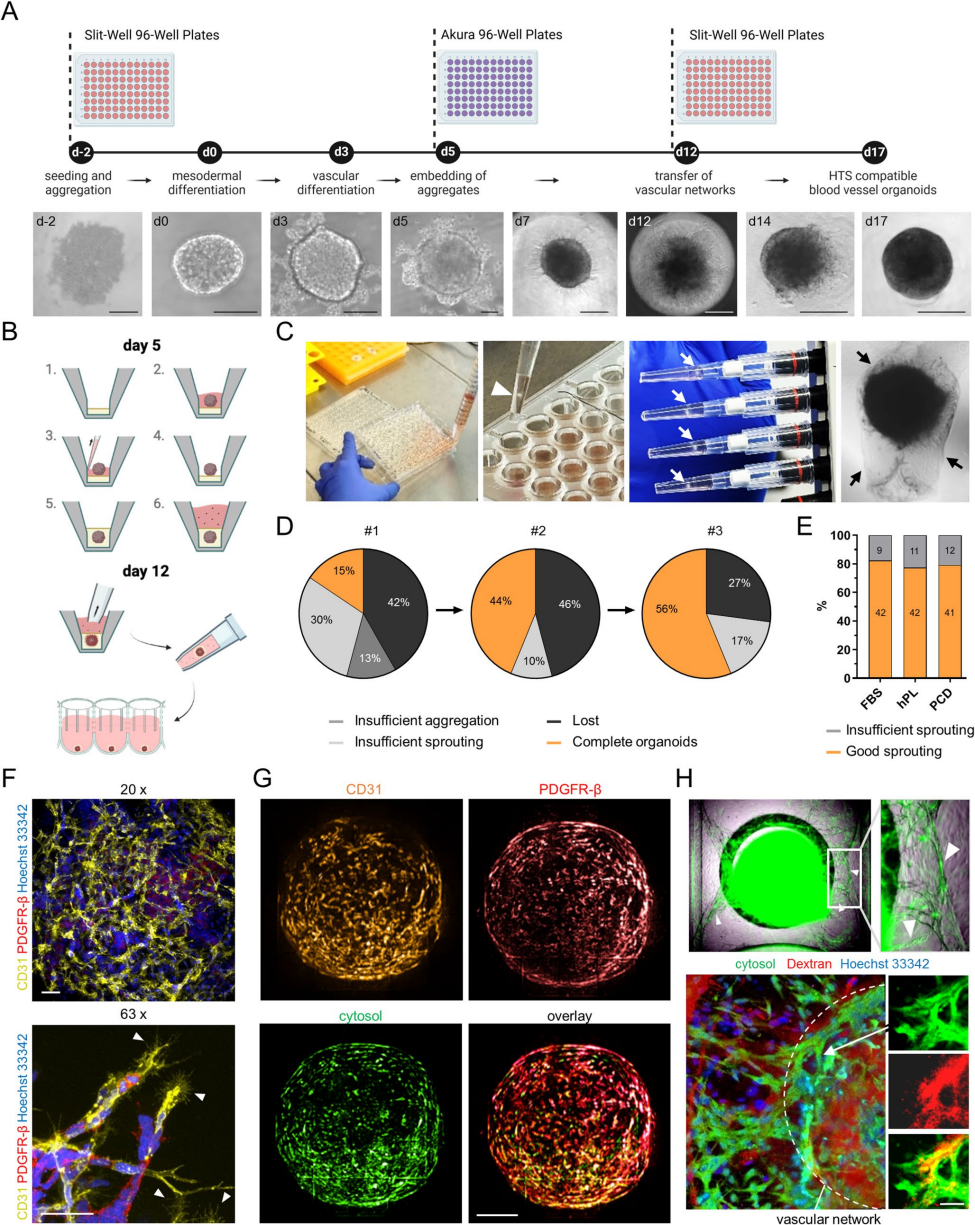
High-throughput (HT)-compatible and nearly xeno-free synthesis of vascular networks and blood vessel organoids from fluorescently tagged human induced pluripotent stem cells (hiPSCs). **A** Schematic illustration of the new differentiation protocol and representative images for the main differentiation steps (scale bars: d-2, d0, d3, d5 = 100 µm; d7, d12 = 250 µm; d14, d17 = 500 µm). Created in BioRender. Skowronek, D. (2025) https://BioRender.com/e70e302. **B** Shown are the steps of embedding the vascular aggregates in an Akura 96-well plate and transferring the vascular networks from the Akura 96-well plate to a PrimeSurface 96 Slit-well plate. **C** The use of PrimeSurface 96 Slit-well plates reduces the time required for medium exchange (left image). Akura 96-well plates allow aggregates to be embedded in small cavities, minimizing the matrix surrounding the vascular networks (black arrows) and allowing direct transfer of vascular networks (white arrows) to new plates without time-consuming manual extraction of the networks from the gel (middle and right images). **D** The new protocol is simple to handle and achieves high synthesis efficiency after minimal training. Shown are the efficiencies of three training runs. **E** The sprouting efficiency is maintained when fetal bovine serum (FBS) is replaced with human platelet lysate (hPL) or chemically defined Panexin CD (PCD). The total numbers of sufficiently sprouted networks and vascular aggregates with insufficient sprouting are written inside the bars. **F,G** HiPSC-derived vascular networks (F) and blood vessel organoids (G) differentiated with the HT-compatible and nearly xeno-free protocol consist of a complex network of endothelial cells (CD31) and associated pericytes (PDGFR-β) [representative images; scale bars: 50 µm (**F**); 200 µm (**G**)]. White arrowheads indicate angiogenic sprouts. **H** Perfusion of vascular networks with TMR-amino-dextran in OrganoPlate graft plates shows anastomoses between the GFP-labeled vascular networks and the HUVEC-derived vascular bed (top, white arrowheads) as well as correct formation and permeability of the vascular networks (bottom, scale bar: 50 µm)

To reduce the workload of blood vessel organoid differentiation and make it more suitable for high-throughput applications, we first modified some key steps (Fig. 1A, B) of the original differentiation protocol [27, 28]. The use of Slit-well plates allowed precise control of the aggregate size while reducing the time spent on media exchange compared to conventional ultra-low attachment 6-well or 96-well cell culture plates. Since all wells in a Slit-well plate share one media pool, medium exchange can be performed using a 25 mL serological pipette (Fig. 1C). Our new protocol also eliminates the most labor-intensive step of blood vessel organoid differentiation, namely cutting out the vascular networks from the collagen I/Matrigel matrix under a sterile bench. Since the embedding of vascular aggregates on day 5 of the protocol takes place in special Akura 96 spheroid microplates, which have a conical well design with a bottom diameter of only 1 mm, the vascular networks can be directly transferred by pipetting to a new 96-well plate after sprouting. This can save several hours of hand-on time per differentiation run. The amount of excess matrix around the vascular networks is minimal (Fig. 1B, C) and easily dissolved by regular pipetting up and down with cut pipette tips over the next seven days. The use of these special cell culture plates makes it relatively easy to learn the new protocol (Fig. 1D).

Besides modifying the workflow, we also adapted the reagents used in the differentiation and replaced bovine collagen I and fetal bovine serum (FBS) with human collagen I and human platelet lysate (hPL) or Panexin CD (PCD), respectively. Since FBS, hPL, and PCD led to comparable sprouting efficiencies (Fig. 1E), we decided to use the chemically defined serum replacement PCD for further experiments. This means that the differentiation protocol is now not only nearly xeno-free, but also significantly less susceptible to lot-to-lot variability. Only Matrigel could not be replaced. Vascular networks and blood vessel organoids generated with the modified protocol are approximately 0.5 to 1.0 mm in size and display highly complex endothelial networks with associated pericytes (Fig. 1F, G). Using the OrganoPlate Graft technology, we were able to demonstrate proper lumen formation in hiPSC-derived vascular networks. After grafting mEGFP-tagged vascular networks onto a HUVEC bed, perfusion was visualized by introducing TMR-amino-dextran (Fig. 1H).

### Perfusion of human blood vessel organoids on chorioallantoic membranes (CAM)

**Fig. 2:**
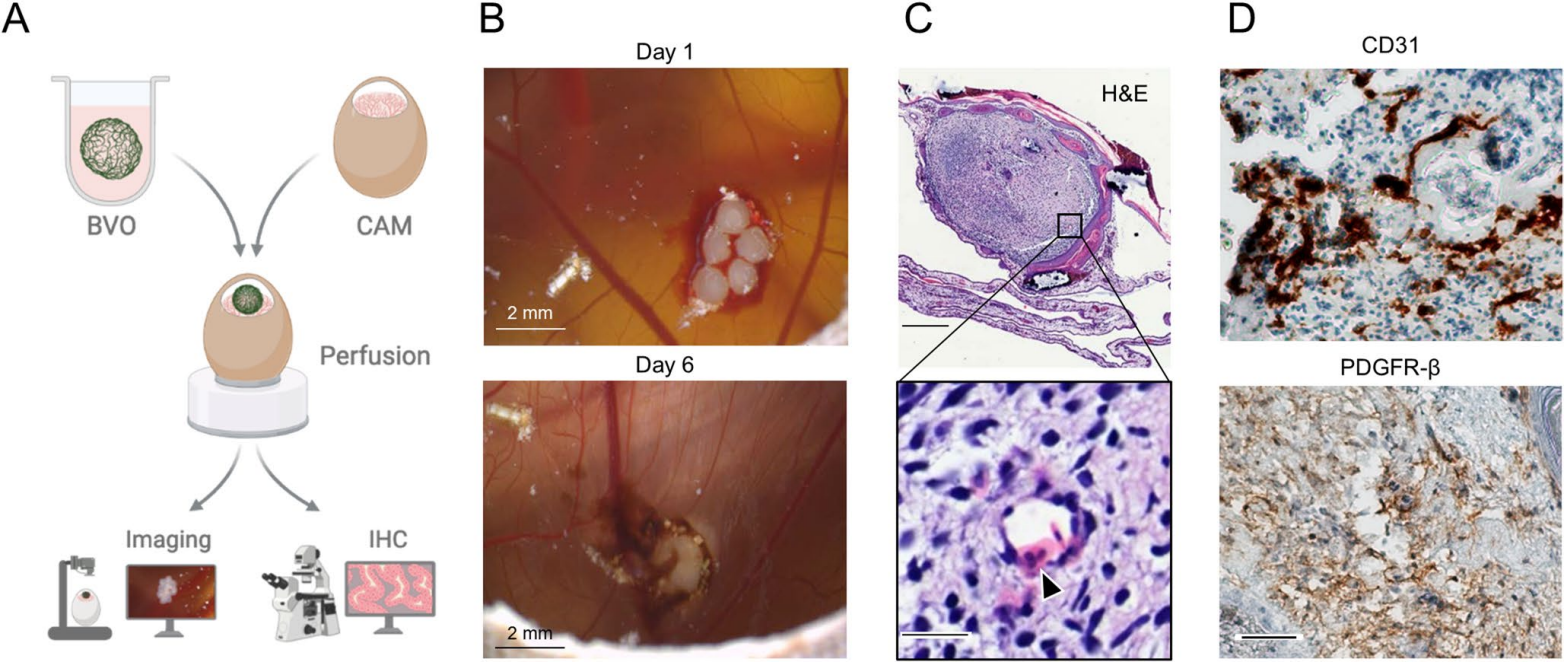
Perfusion of blood vessel organoids (BVO) on chorioallantoic membranes (CAM). **A** Schematic illustration of the perfusion approach. Created in BioRender. Skowronek, D. (2025) https://BioRender.com/n06n764.** B** Shown are blood vessel organoids cultivated on the CAM associated with chicken blood vessels. The pictures were taken on day 1 and day 6 (scale bars = 2 mm). **C** Sectioning and H&E staining demonstrated nucleated chicken erythrocytes within the vascular structures of the blood vessel organoid (black arrow head) (upper scale bar = 500 µm; bottom scale bar = 25 µm). **D** The expression of CD31 (upper image) and PDGFR-β (lower image) were verified by immunohistochemistry staining (brown) (scale bar = 100 µm)

As a next step, we wanted to combine our easy to use, scalable, and almost xeno-free blood vessel organoid differentiation protocol with the chorioallantoic membrane (CAM) 3D in vivo model, which is commonly used for perfusion approaches [45, 50]. One week after grafting onto the CAM, the blood vessel organoids were explanted for hematoxylin and eosin (H&E) staining and immunohistochemical staining for CD31 and PDGFR-β (Fig. 2A–D). We found intact endothelial and pericyte structures in the grafted blood vessel organoid sections. To differentiate between chicken and human blood vessels, an antibody that specifically targets human CD31 (PECAM1) was used. In H&E-stained tissue sections, nucleated chicken erythrocytes were found within the vascular structures of the blood vessel organoids, indicating successful perfusion in the CAM model (Fig. 2C).

### Irregular aggregation of hiPSCs, mesodermal, and vascular endothelial cells upon CCM1 and CCM3 inactivation

**Fig. 3:**
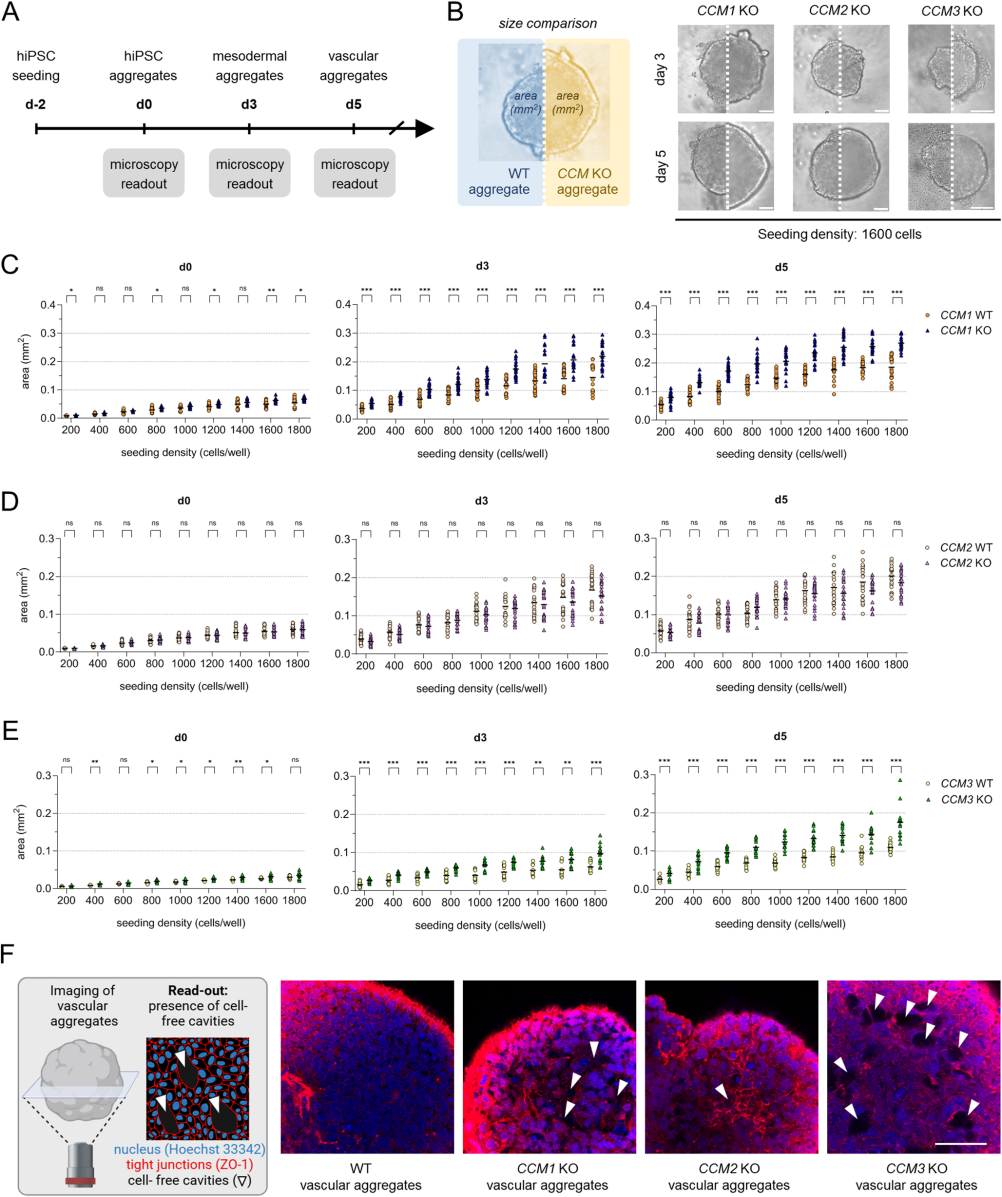
Size differences between WT and KO aggregates. **A** Schematic illustration of the initial hiPSC aggregation and the induction of mesodermal and vascular differentiation. Created in BioRender. Skowronek, D. (2025) https://BioRender.com/c48w302. **B** Shown are representative images of wild-type (WT; left half of each image) and *CCM1*, *CCM2,* and *CCM3* knockout aggregates (KO; right half of each image) on days 3 and 5 (initial seeding density: 1,600 cells/well, scale bars: 100 µm). **C-E** WT and *CCM1* (C), WT and *CCM2* (D), WT and *CCM3* (E) KO hiPSCs were seeded with variable seeding densities (200 to 1,800 cells/well) at d-2. The cross-sectional area of the aggregates was determined on days 0, 3, and 5. Shown are individual data points, line represents mean, n = 12–24 per genotype in three independent biological replicates. Multiple two-sample t-tests with Welch's correction and Holm-Šídák adjustment for multiple testing were used for statistical analyses (ns = Padj ≥ 0.05; * = Padj < 0.05; ** = Padj < 0.01; *** = Padj < 0.001). **F** Staining of tight junction protein 1 (= zonula occludens protein 1/ZO-1) revealed cell free cavities (white arrows) in *CCM1* and *CCM3* KO vascular aggregates (scale bars: 50 µm)

To further elucidate the pathobiology of CCM in a human blood vessel organoid model, we first generated *CCM1* and *CCM2* KO hiPSCs from the mEGFP-tagged AICS-0036-006 hiPSC line using CRISPR/Cas9 genome editing and limiting dilution cloning (Fig. S1). The generation of mEGFP-tagged *CCM3* KO AICS-0036-006 hiPSCs had already been described in our previous publication [30]. Already the initial steps of differentiation of KO- and WT-hiPSCs into vascular organoids revealed significant differences (Fig. 3A, B). *CCM1* KO and *CCM3* KO aggregates were significantly larger than the wild-type controls. These differences were observed at various cell seeding densities and became more pronounced over time at the mesodermal (day 3) and vascular differentiation (day 5) stages (Fig. 3B, C, E). In contrast, no significant size differences were found for *CCM2* KO aggregates (Fig. 3B, D). To exclude proliferation differences in KO hiPSCs as a factor in size variation, we performed proliferation studies, which demonstrated no significant differences between KO and WT hiPSCs (Fig. S2). However, immunofluorescence staining for tight junction protein 1 (ZO-1) revealed large cell-free cavities in *CCM1* and *CCM3* KO vascular aggregates, while these were rarely observed in WT or *CCM2* KO aggregates (Fig. 3F). Consistent with previous studies [37, 51–53] these findings suggest that impaired cell–cell junctions may drive the observed size differences.

### Structural defects in KO vascular networks

**Fig. 4:**
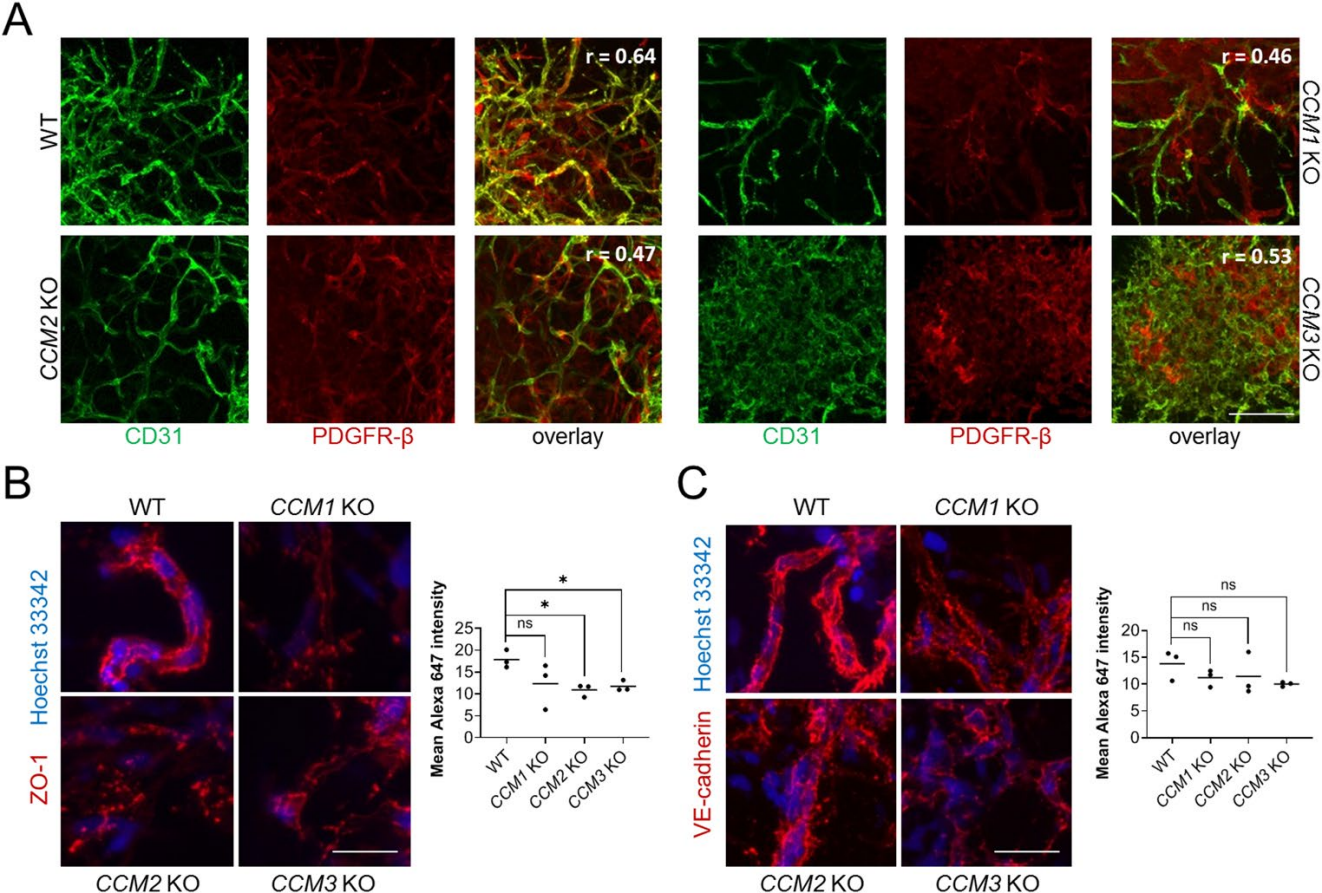
Structural defects in KO vascular networks. **A** Immunofluorescence staining for CD31 (endothelial marker, green) and PDGFR-β (pericyte marker, red) indicated a reduced correlation between ECs and pericytes in *CCM1, CCM2,* and *CCM3* KO vascular networks (scale bar: 100 µm). Correlation was evaluated by using the Pearson’s correlation coefficient (r) calculated with the JACoP ImageJ plugin. **B**, **C** Immunofluorescence staining for ZO-1 (B) and VE-cadherin (**C**) revealed irregular tight and adherens junctions in *CCM1*, *CCM2,* and *CCM3* KO vascular networks (Alexa 647; scale bars: 25 µm). Statistical analyses demonstrated a significant reduction of Alexa 647 (ZO-1) fluorescence intensity in *CCM2* and *CCM3* KO networks. Data are presented as individual data points and means. Multiple two-sample t-tests with Welch's correction and Holm-Šídák adjustment for multiple testing were used for statistical analyses (* = Padj < 0.05)

We next focused on the stage of vascular network formation. Immunofluorescence analyses showed reduced co-localization of the endothelial marker CD31 and the pericyte marker PDGFR-β, indicating a weaker association of ECs and pericytes in KO vascular networks (Fig. 4A). In *CCM3* KO samples, we also saw more convoluted endothelial and pericyte networks. Furthermore, the formation of tight and adherens junctions was impaired in *CCM1*, *CCM2*, and *CCM3* KO vascular networks. While we found a continuous linear pattern of ZO-1 and VE-cadherin in the WT vascular networks, an irregular, more punctate pattern was seen in the three KO conditions (Fig. 4B, C). Although ZO-1 and VE-cadherin localization was highly disorganized, overall expression appeared to be less affected. We found significantly reduced ZO-1 intensity in *CCM2* and *CCM3* KO vascular networks, but only trends in the other comparisons (Fig. 4B, C).

### Altered cellular composition in KO blood vessel organoids

**Fig. 5:**
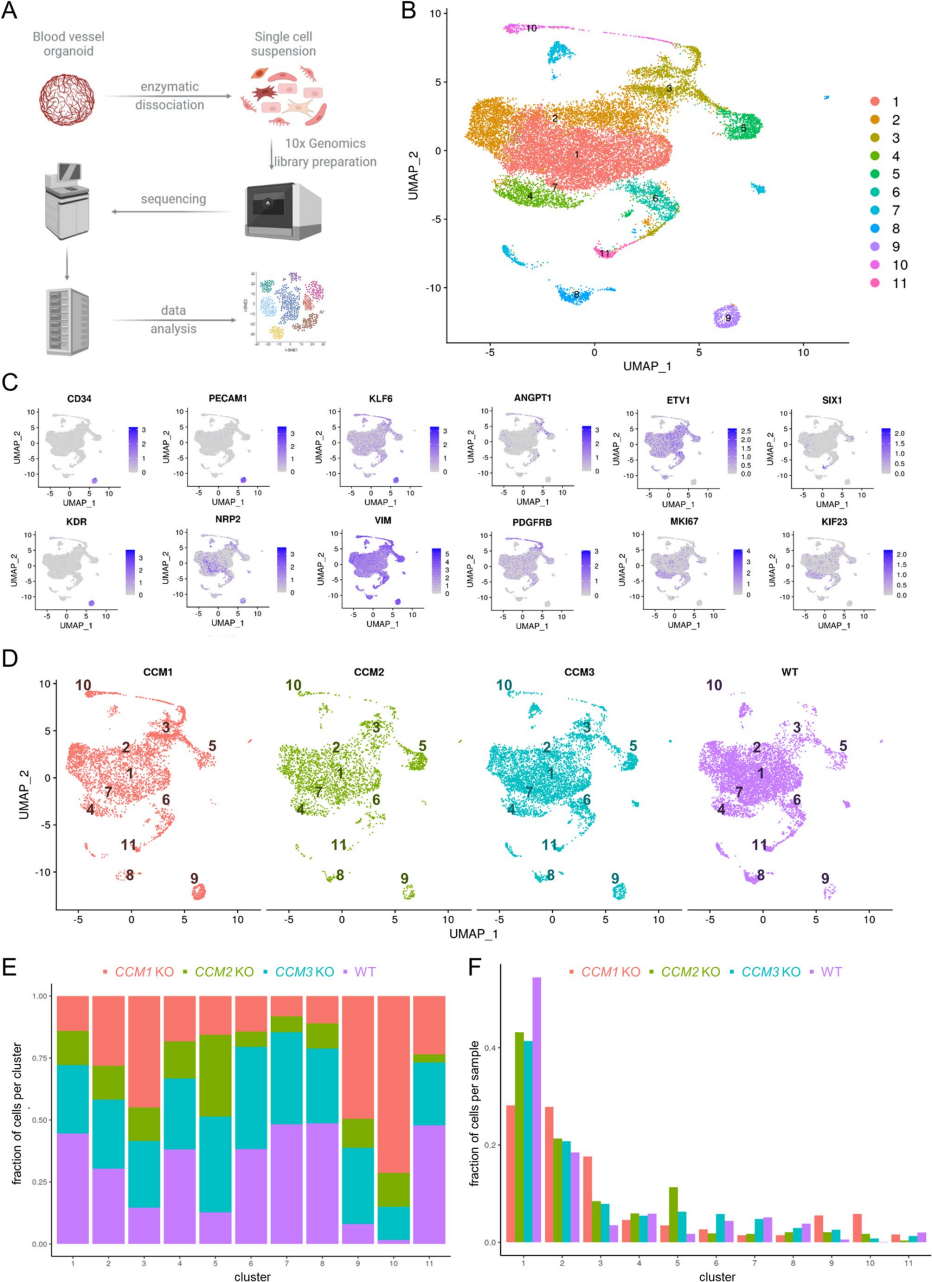
Changes in cellular composition of KO blood vessel organoids. **A** Experimental design of the single-cell RNA sequencing (scRNA-seq) experiments for KO and WT organoids. Created in BioRender. Skowronek, D. (2025) https://BioRender.com/buao0as. **B** Uniform Manifold Approximation and Projection (UMAP) visualization of the scRNA-seq data with cells colored according to the unsupervised clustering results.** C** UMAP plots colored by the expression levels of marker genes predominantly expressed in endothelial, pericyte, smooth muscle and proliferating cells. Purple color indicates high expression. Low expression is indicated by grey color. **D** Separate UMAP plots of scRNA-seq data from *CCM1* KO, *CCM2* KO, *CCM3* KO, and WT blood vessel organoids. The numbers represent the different clusters. **E** The composition of the clusters is illustrated by the proportion of *CCM1* KO, *CCM2* KO, *CCM3* KO, and WT cells within each cluster. **F** The cellular composition of KO and WT blood vessel organoids is illustrated by the distribution of *CCM1* KO, *CCM2* KO, *CCM3* KO, and WT samples into the different clusters

To further characterize the cellular composition and genotype-specific gene expression differences in WT and KO blood vessel organoids, we used scRNA-seq analysis (Fig. 5A). After integrating the datasets and unsupervised clustering, we identified 11 clusters with unique gene expression signatures. The visualization in an UMAP plot demonstrated good separation of the clusters (Fig. 5B). By analyzing cell-type-specificity of marker genes using the WebCSEA tool, we identified clusters of ECs (c1, c9), perivascular/stromal cells (c3, c5, c6, c10), cells with neuronal and glial gene expression signature (c7, c8, c11), and proliferating cells (c4) (Fig. 5B, C and S4). Cluster specific marker genes were, for example, *PECAM1*, *KDR,* and *CD34* (EC cluster c9), *SIX1* (neuronal-like cluster c11) as well as *MKI67* and *KIF23* (proliferating cell cluster c4) (Fig. 5C). Perivascular/stromal cell clusters were characterized by fibroblastic, mesenchymal, and smooth muscle cell gene expression signatures (Fig. S4). High expression of *PDGFRB* was observed in cluster c10 indicating the presence of pericytes (Fig. 5C). Inactivation of *CCM1*, *CCM2,* or *CCM3* resulted in significant shifts in the cellular composition of the organoids. While there were many overlapping effects, comparative analysis also revealed genotype-specific differences (Fig. 5D–F). For example, *CCM1* KO organoids had a marked over-representation of cells in clusters c3, c9, and c10. In contrast, *CCM2* KO organoids showed a remarkably high proportion of cells in cluster c5. Changes in cellular composition were also observed in *CCM3* KO organoids. In particular, the fraction of cells in clusters 6 and 7 were similar to the WT condition, while they were underrepresented in *CCM1* and *CCM2* KO organoids.

### Differential gene expression in KO blood vessel organoids

**Fig. 6:**
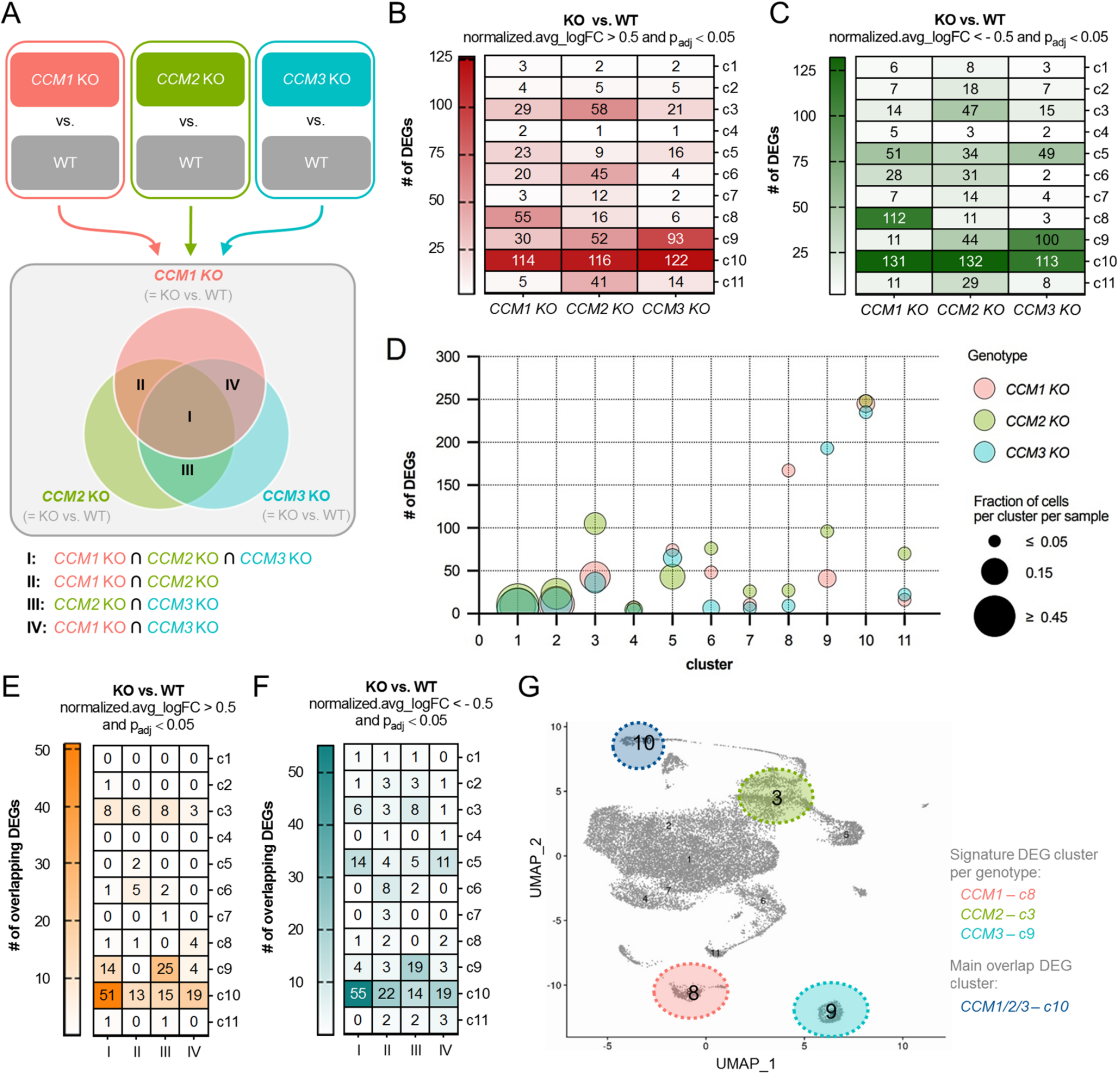
Overlapping and genotype-specific gene expression differences in KO blood vessel organoids. **A** Scheme of the analysis strategy and the selected comparisons. **B,C** Heatmaps with the numbers of significantly up- (B) and downregulated (C) genes per cluster and genotype. DEGs** = **differentially expressed genes. **D** The relationship between the total number of DEGs per genotype and cluster and the fraction of cells per cluster in the *CCM1*, *CCM2,* and *CCM3* KO samples is illustrated in a bubble plot.** E**, **F** Heatmaps with the numbers of overlapping up- (E) and downregulated (F) DEGs between the different genotypes. I = *CCM1* KO ∩ *CCM2* KO ∩ *CCM3* KO; II = *CCM1* KO ∩ *CCM2* KO; III = *CCM2* KO ∩ *CCM3* KO; IV = *CCM1* KO ∩ *CCM3* KO.** G** The main overlap DEG cluster (c10) and the signature DEG clusters per genotype (*CCM1* KO: c8; *CCM2* KO: c3; *CCM3* KO: c9) are highlighted in the UMAP plot. Significantly up- and downregulated genes were defined as those with a normalized.avg_logFC (KO vs. WT) > 0.5 and *p*_adj_ < 0.05 or with a normalized.avg_logFC (KO vs. WT) < -0.5 and *p*_adj_ < 0.05, respectively

**Fig. 7:**
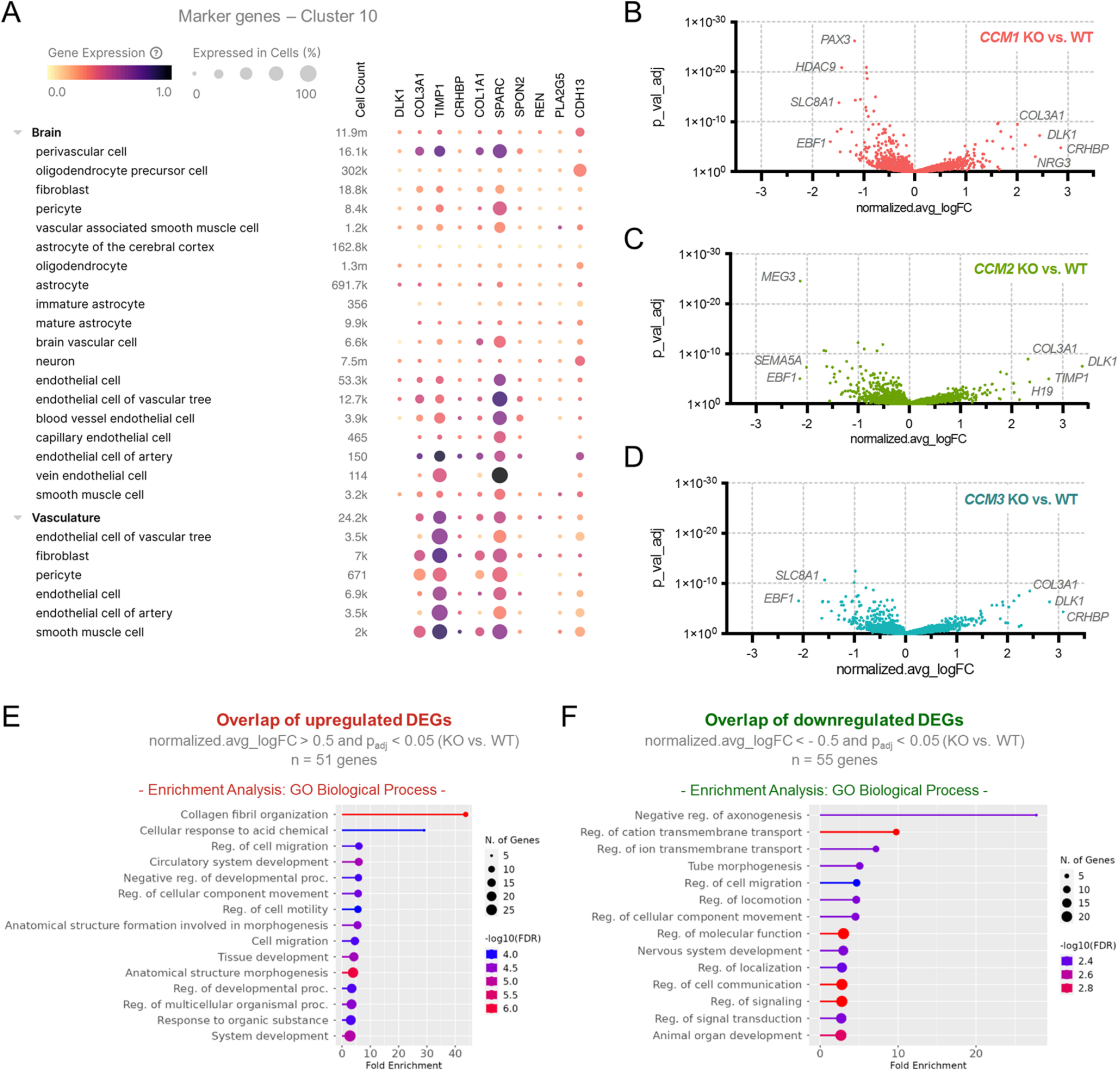
Overlapping gene expression differences in cluster 10.** A** The CZ CELLxGENE Discover browser was used to visualize the tissue and cell type-specific expression levels of the top 10 marker genes identified in cluster 10. Shown are cell types that are typically found in the brain and the vasculature. Purple color indicates high expression. Low expression is indicated by yellow color. The percentage of cells of the specific cell type that express the marker gene is visualized by the size of the circles. **B**–**D** Genotype-specific gene expression differences in cluster 10 are shown in Volcano plots for *CCM1* (B), *CCM2* (C), and *CCM3* (D) KO samples. **E**, **F** Overlapping up- (**E**) and downregulated (**F**) genes were subjected to gene set enrichment analyses with the GO biological process gene set. Significantly up- and downregulated genes were defined as those with a normalized.avg_logFC (KO vs. WT) > 0.5 and p_adj_ < 0.05 or with a normalized.avg_logFC (KO vs. WT) < -0.5 and p_adj_ < 0.05, respectively. Overlapping DEGs were defined as DEGs found in *CCM1*, *CCM2,* and *CCM3* KO samples (= *CCM1* KO ∩ *CCM2* KO ∩ *CCM3* KO)

Overlapping and genotype-specific effects were also observed at the gene expression level (Fig. 6A). Clusters c3, c5, c6, c8, c9, and c10 showed the highest numbers of differentially expressed genes (DEGs) per genotype (Fig. 6B–D). Indeed, cluster c10 had the highest number of DEGs (Fig. 6B–D). Since this cluster also showed the largest overlap of DEGs between the three KO conditions (Fig. 6E, F), it was classified as the main overlap cluster (Fig. 6G). The genes that were most up- or downregulated in this fibroblast cell cluster (Fig. 7A and S4) were *EBF1*, *COL3A1,* and *DLK1*. These genes were similarly deregulated in all three KO genotypes (Fig. 7B–D). A GO ontology analysis of the 51 genes significantly upregulated in all three KO conditions showed a strong enrichment for genes that are involved in collagen fibril organization (Fig. 7E). A pathway analysis for the 55 DEGs downregulated in all three KO conditions revealed a more diffuse picture with weaker enrichment for different biological processes (Fig. 7F).

In the other clusters, the numbers of overlapping DEGs were significantly lower. We even found genotype-specific signatures in certain clusters. For example, only *CCM1* KO organoids had a high number of DEGs in cluster c8. Similar characteristic DEG clusters were also found in *CCM2* and *CCM3* KO organoids. Here, specific gene expression differences were found in clusters c3 and c9, respectively (Fig. 6B, C, G). In the EC cluster c9, a total of 193 DEGs were found in the *CCM3* KO organoids, but only 41 and 96 in *CCM1* and *CCM2* KO organoids, respectively. Only 18 DEGs were found to overlap in all three KO genotypes including *KLF2*, *ESM1*, *COL3A1*, *VEGFC*, *NRP2*, *AKAP12*, *S100A4*, *IGFBP5,* and *HYAL2* (Fig. S5A,B). These were mainly associated with angiogenesis-related processes (Fig. S5C). There was additional overlap between *CCM2* and *CCM3* KO organoids but hardly any between *CCM1* and *CCM2* or *CCM1* and *CCM3* KO organoids (Fig. 6E,F). The *CCM3*-specific DEGs in this cluster (Fig. S5D,E) mainly included genes involved in cell–matrix adhesion (Fig. S5F,G). We also detected expression changes in key genes commonly deregulated in CCM disease (Table S1). Notably, *KLF2* was significantly upregulated in all three genotypes. Other targets, including *KLF4*, *THBD*, and *RHOA*, exhibited genotype-specific deregulation, with the strongest effects observed in *CCM3* KO ECs. These results provide molecular validation for our model, reinforcing its relevance in light of existing findings.

In the neuronal-like cell cluster c8 (Fig. S6A), a total of 167 DEGs were found in the *CCM1* KO organoids, whereas only few genes were deregulated in the *CCM2* and *CCM3* KO condition (Fig. S6B-D). Only the two genes *TMSB4X* and *GRM8* were commonly deregulated in all three KO genotypes (Fig. S6E,F). The *CCM1*-specific DEGs in this cell cluster were associated with axonogenesis and neuron differentiation (Fig. S6G). In the mesenchymal-like cell cluster c3 (Fig. S7A), the highest number of DEGs was found in *CCM2* KO organoids, with 14 DEGs overlapping with the *CCM1* and *CCM3* KO genotype (Fig. S7B,C). The *CCM2*-specific DEGs were associated with mesenchyme development and regulation of cell motility (Fig. S7). Interestingly, in *CCM3* KO organoids, the clusters c6 and c7, for which the fraction of cells was similar to WT organoids, also showed a very low number of DEGs compared to *CCM1* and *CCM2* KO conditions. This indicates less pronounced effect of CCM3 deficiency in cells of the perivascular/stromal (c6) as well as neuronal- and glial-like (c7) cell clusters.

### Abnormal proliferation of KO cells in mosaic blood vessel organoids and endothelial co-cultures

**Fig. 8:**
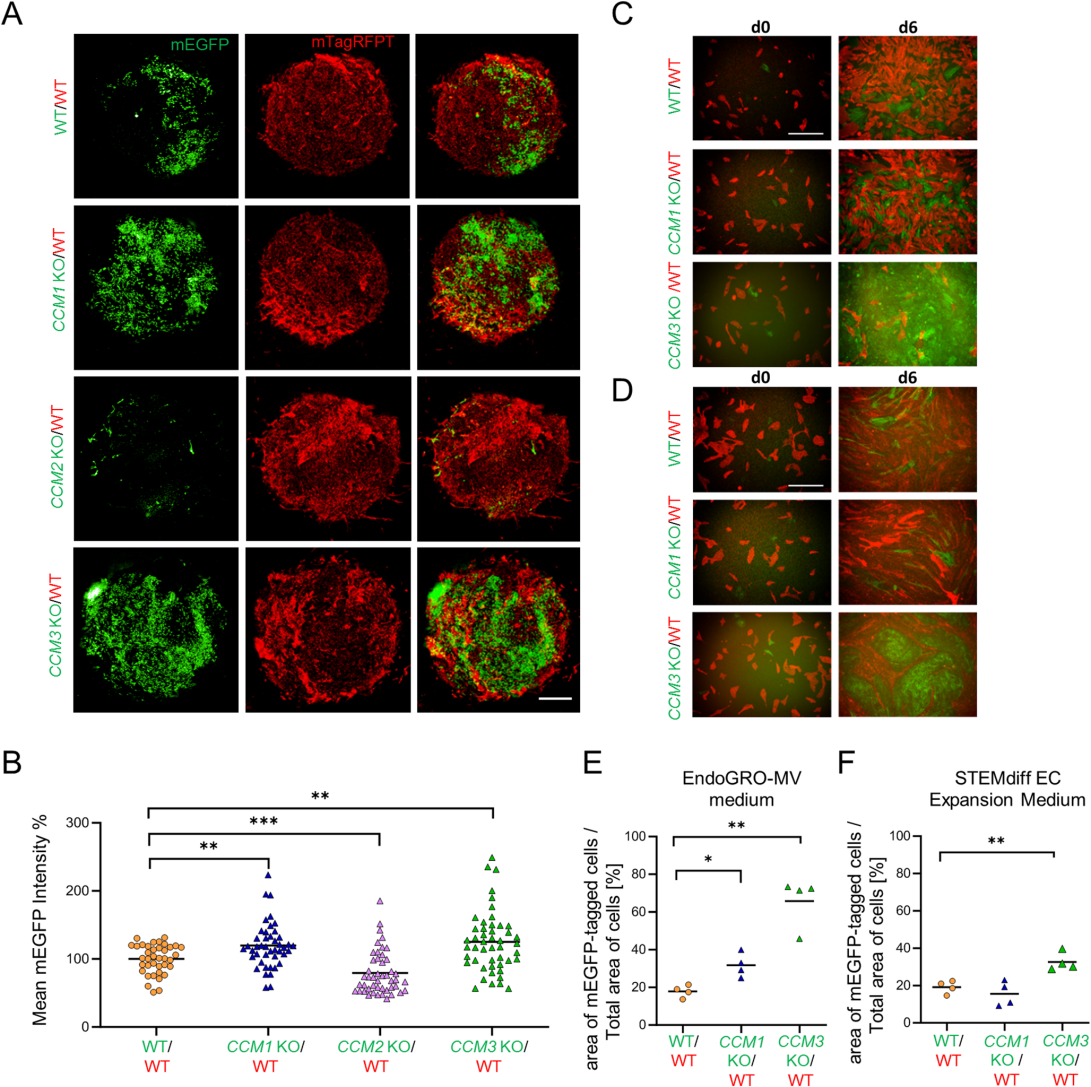
Abnormal proliferation of KO cells in mosaic blood vessel organoids and EC co-cultures.** A** In mosaic blood vessel organoids consisting of *CCM1* KO and wild-type cells (= *CCM1* KO/WT) or *CCM3* KO and wild-type cells (= *CCM3* KO/WT), abnormally increased proliferation of *CCM1* KO and *CCM3* KO cells was observed. In mosaic blood vessel organoids consisting of *CCM2* KO and wild-type cells (= *CCM2* KO/WT), a reduced proliferation of *CCM2* KO cells was found. KO cells were labeled with mEGFP. WT cells were labeled either with mTagRFPT or mEGFP (scale bar: 200 µm). **B** Mean mEGFP intensities in mosaic organoids consisting of mEGFP-labeled KO and mTagRFPT-labeled WT cells (= KO/WT) were normalized to the mean mEGFP intensity in control mosaic organoids consisting of mEGFP-labeled WT and mTagRFPT-labeled WT cells (= WT/WT). Mosaic blood vessel organoids were differentiated in three independent runs (n = 38–48 per genotype). Data are presented as individual data points and means. Statistical significance was assessed using the Mann–Whitney U-test with Welch's correction (**P < 0.01, ***P < 0.001). **C**, **D** In a 2D co-culture validation approach, mEGFP-labeled *CCM1* or *CCM3* KO iECs and mTagRFPT-labeled WT iECs were seeded in a 1:9 ratio (= *CCM1* KO/WT or *CCM3* KO/WT) and cultivated in either EndoGRO-MV (C) or STEMdiff EC expansion medium (D). Co-cultures of mEGFP-labeled WT and mTagRFPT-labeled WT iECs (= WT/WT) served as controls (scale bar: 200 µm). **E**, **F** After 6 days, the area of mEGFP-labeled cells compared to all cells was determined using FIJI software (n = 4 per genotype in three independent biological replicates). KO/WT = co-cultures of mEGFP-labeled KO and mTagRFPT-labeled WT iECs; WT/WT = co-cultures of mEGFP-labeled WT and mTagRFPT-labeled WT iECs. Data are presented as individual data points and means. Multiple two-sample t-tests with Welch's correction and Holm-Šídák adjustment for multiple testing were used for statistical analyses (* = Padj < 0.05; ** = Padj < 0.01)

We have previously demonstrated that *CCM3* KO cells show an abnormally high proliferation in *CCM3* KO/WT mosaic blood vessel organoids [30]. With our new differentiation protocol, we not only reproduced our previous result but also studied the behavior of *CCM1* KO and *CCM2* KO cells in mosaic blood vessel organoids. KO hiPSCs labeled with mEGFP were mixed with mTagRFPT-labeled WT hiPSCs and differentiated into mosaic blood vessel organoids. In *CCM3* KO/WT and *CCM1* KO*/*WT mosaic blood vessel organoids, significantly increased mEGFP signals were observed when compared to control mosaic blood vessel organoids that had been differentiated from a mix of mEGFP- and mTagRFPT-labeled WT cells (Fig. 8A, B). In *CCM2* KO/WT mosaic organoids, on the other hand, we saw significantly lower mEGFP signals (Fig. 8A, B). To better understand the interaction of KO and WT cells, we synthesized mosaic vascular networks and performed immunofluorescence staining. In these analyses, CD31 positive endothelial and PDGFR-β positive pericyte networks appeared more convoluted in *CCM3* KO mosaic vascular networks than in *CCM1* and *CCM2* KO mosaic networks (Fig. S8A). Notably, in mosaic *CCM3* KO/WT vascular networks, we also observed many KO cells lacking proper VE-cadherin expression (Fig. S8B and Supplementary Videos 1–4).

To confirm the observed proliferative advantage of *CCM3* KO and *CCM1* KO cells in mosaic blood vessel organoids, we went back to a two*-*dimensional hiPSC-derived EC (iEC) co-culture assay. Immunofluorescence staining of endothelial markers confirmed the successful differentiation of hiPSCs into iECs (Fig. S3A). In line with published literature, KO iECs showed remodeling of the actin cytoskeleton with stress fiber formation (Fig. S3B) [54–56]. Afterwards, we mixed mTagRFPT-labeled WT iECs with mEGFP-labeled *CCM1* or *CCM3* KO iECs in a 9:1 ratio. After 6 days, mEGFP signals were significantly increased in KO/WT co-cultures. In particular, *CCM3* KO iECs exhibited markedly enhanced, almost "tumor-like" proliferation, which was most prominent in EndoGRO-MV medium (Fig. 8C, E). In STEMdiff endothelial expansion medium, the proliferation of *CCM3* KO iECs was also significantly increased, but the effect was less pronounced (Fig. 8D, F). Compared to *CCM3* KO iECs, *CCM1* KO iECs co-cultured with WT iECs in EndoGRO-MV medium demonstrated a more modest proliferative advantage (Fig. 8C, E). In STEMdiff medium, no proliferation difference was found for *CCM1* KO iECs (Fig. 8D, F). Although the immunofluorescence analysis of mosaic vascular networks was performed with another focus, a different proliferative behavior was also evident in this setting, with an increased number of KO cells observed especially in the *CCM3* KO/WT condition (Fig. S8B and Supplementary Videos). Taken together, these data show that inactivation of *CCM1* or *CCM3* alone does not induce hyperproliferation but can trigger an abnormal proliferation in a specific microenvironment.

## Discussion

Although the differentiation of blood vessel organoids is still a relatively new cell culture technique [27], it already had a significant impact on endothelial and vascular biology research in recent years [28, 57–59]. However, one limitation of the protocol has been its lack of scalability. In addition, transplantation into mice was usually required to perfuse the organoids [28, 60]. In this study, we have simplified the workflow and present a high-throughput compatible protocol fully optimized for a 96-well format. Together with the use of a chemically defined, animal-free serum replacement and the possibility to perfuse the organoids or vascular networks on a CAM or in an OrganoPlate Graft plate, the new protocol facilitates the replacement of animal studies in biomedical research. The two perfusion models from our study can complement another recently described in vitro perfusion approach using a sophisticated microfluidic device [61] so that both simple and more complex perfusion experiments can now be realized without transplantation into mice. Since the vascularization and perfusion of organoids is also a high priority in other fields of biomedical research [62–64], these results may have an impact beyond vascular medicine.

Our data also demonstrate that blood vessel organoids are excellent tools for studying CCM disease. While mouse models have provided invaluable insights into key mechanisms of CCM pathogenesis, e.g., deregulation of MEKK3-KLF2/4 signaling, increased endothelial activation of PI3K-mTOR signaling, loss of junctional integrity, clonal expansion of KO ECs, or recruitment of WT cells into growing CCM lesions [17, 31, 32, 65–68], they can hardly be used for a comprehensive comparison between *CCM1*, *CCM2,* and *CCM3* KO conditions. In accordance with the 3R principle (Replacement, Reduction, Refinement) [69], blood vessel organoids now allow the direct comparison of the cellular and molecular consequences of *CCM1*, *CCM2,* and *CCM3* gene inactivation in a complex three-dimensional cell culture system. Differences between the genotypes were already evident at the stages of mesodermal and vascular aggregates. In particular, the *CCM1* and *CCM3* KO aggregates were significantly larger than the WT and *CCM2* KO aggregates and also had large cavities inside. The genotype-specific differences are reminiscent of results from *Ccm*^*BECKO*^ mice, where most severe CCM lesions were found in *Ccm3*^*BECKO*^ mice, followed *Ccm1*^*BECKO*^ and *Ccm2*^*BECKO*^ mice [70]. However, it is also possible that endothelial dysfunction will develop with different dynamics upon *CCM1*, *CCM2,* or *CCM3* inactivation. In line with this hypothesis, we found defects in tight and adherens junctions also in *CCM2* KO samples at later stages of vascular network differentiation.

We have recently demonstrated tumor-like proliferation of *CCM3* KO cells in mosaic KO/WT blood vessel organoids [30], a behavior also described for *Ccm3* mouse models [31, 32]. Here, we have reproduced this effect with our new protocol and found a similar phenomenon in mosaic *CCM1* KO/WT organoids. Previously, there was evidence from human blood outgrowth endothelial cells and also from mice that inactivation of the *CCM1*/*Ccm1* gene can enhance EC proliferation [33, 34], while other reports suggested that *CCM1* silencing impairs endothelial cell growth [38]. While the latter observation seems to contradict the results of our mosaic organoid experiments, our data also show that the proliferation advantage of *CCM1* and *CCM3* KO cells is not an inherent effect of the gene inactivation but can be induced in a specific microenvironment. Indeed, different media significantly modulated the tumor-like proliferation of KO iECs in KO/WT co-cultures. *CCM1* KO iECs proliferated abnormally in EndoGRO-MV medium, but not in STEMdiff medium. A less pronounced but still significant increase in cell growth was also observed for *CCM3* KO iEC in STEMdiff medium. These results highlight the critical role of growth factors and local stress events, such as oxidative stress and inflammatory stimuli [71], in the development of the endothelial dysfunction in CCM1- and CCM3-deficient cells, which may represent a therapeutic target.

Interestingly, *CCM2* KO cells displayed altered proliferation dynamics in mosaic KO/WT vessel organoids and showed impaired cell growth. Consistent with this observation, Vannier and colleagues found a decreased proliferation rate following siRNA-mediated *CCM2* gene silencing in HUVECs [38]. However, other studies found no effect of CCM2 inactivation on proliferation, suggesting a context-specific effect [72]. A possible explanation for the lack of increased proliferation in *CCM2* KO cells is the presence of CCM2L, a paralog of CCM2 that can partially compensate for its loss. In vivo, CCM2L cannot completely block CCM formation in mice with endothelial-specific *Ccm2* deletion (*Ccm2*^*iECKO*^) but prevents a more severe phenotype that would result from a double knockout of *Ccm2* and *Ccm2l* [73]. At the molecular level, this compensatory effect is mediated by a partial blockade of MEKK3 signaling by CCM2L [73, 74]. Since MEKK3-KLF2/4 signaling regulates endothelial proliferation and survival [66, 75, 76], its inhibition by CCM2L may explain the different behavior of *CCM2* KO cells.

Further insights into the functions of the three CCM proteins were also gained from our scRNA-seq data. As might be expected from the fact that cavernous lesions show major morphological and functional defects but no complete disruption of the vascular identity itself, we found only moderate effects on the cellular composition of KO organoids. However, at the level of differential gene expression, the overlapping and gene-specific differences were much more pronounced. In particular, deregulation of angiogenesis-related processes was a common feature found in EC cluster 9 of all three KO genotypes. Several genes that were consistently deregulated in ECs, including *ESM1*, *VEGFC*, *NRP2*, *IGFBP5*, and *AKAP12,* are known to play important roles in vessel development. Knockout of the endothelial tip cell marker gene *Esm1* in mice, for example, lead to delayed vascular outgrowth, reduced filopodia extension, and decreased VEGF-induced vascular permeability [77]. Increased VEGF-C/VEGFR3 or NRP2 signaling was observed to contribute to lymphatic defects driven by oncogenic *PIK3CA* mutation [78] or after *Ccm3* silencing [79]. It has also been shown that IGFBP-5 has an antiangiogenic effect in HUVECs [80] and that *AKAP12* deficiency impairs VEGF-induced endothelial cell migration and sprouting in human ECs and mice [81]. Another consistently upregulated gene in EC cluster 9 was *KLF2*, highlighting the pivotal role of augmented MEKK3-KLF2/4 signaling in CCM disease that leads to profound endothelial dysfunction and aberrant angiogenesis via, for example, increased Rho/ROCK and PI3K-mTOR signaling or endothelial-to-mesenchymal transition (EndMT) [17, 66, 68, 82]. The important role of EndMT is highlighted by the upregulation of the EndMT marker genes *S100A4* and *COL3A1* in EC cluster 9 of all three KO genotypes. Upregulation of hyaluronidase HYAL2, previously reported in CCM1-deficient HUVECs [83], further suggests extracellular matrix remodeling as a common pathological feature of KO ECs. Moreover, the upregulation of *KLF4* and *THBD* as well as the downregulation of *CLDN5* in *CCM3* KO ECs closely aligned with findings from a recent scRNA-seq study in a CCM3-deficient mouse model [84], further validating our organoid model.

However, an advantage of the CCM blood vessel organoid model is that it also incorporates the role of non-ECs. Indeed, in all three KO conditions, we identified many overlapping DEGs in the fibroblast cell cluster, which showed a strong enrichment of genes related to collagen fibril organization. Increased expression of ECM genes in *CCM3* KO human brain microvascular pericytes and augmented deposition of fibronectin and collagen in mice with mural cell-specific deletion of *Ccm3* (*Ccm3*^smKO^) have previously been shown to contribute to the dissociation of ECs and pericytes in CCM lesions [85]. However, there also seems to be a cell non-autonomous regulation of ECM production by pericytes in CCM disease. For example, a study in mice with endothelial-specific deletion of *Ccm1* (*Ccm1*^*ECKO*^) revealed transforming growth factor-beta (TGFβ)-mediated upregulation of ECM genes in pericytes and increased fibronectin deposition in CCM lesions [86]. Interestingly, gene expression data for the fibroblast cell cluster also hint to a tumor-like nature of CCMs. For example, *DLK1* was one of the most upregulated genes for all KO genotypes and was previously shown to be a tumor pericyte-associated antigene [87].

The use of blood vessel organoids also revealed non-overlapping effects of *CCM1*, *CCM2*, and *CCM3* inactivation, potentially explaining differences in disease severity among patients with pathogenic variants in these genes. While the aforementioned lack of tumor-like proliferation of *CCM2* KO cells may explain the milder course in patients with a *CCM2* mutation [88], the high number of *CCM3*-specific DEGs in ECs indicates a more profound endothelial dysfunction, consistent with a more severe clinical phenotype in patients with a *CCM3* mutation [89, 90]. Finally, the high number of *CCM1*-specific DEGs in a cell cluster with a neuronal-like gene expression signature gives a first idea of why seizures are the most common manifestation in patients with a *CCM1* mutation [88]. Although CCM1 is expressed in human neurons [91], future studies will have to show whether the gene expression differences are caused by cell-autonomous or non-autonomous mechanisms. The recently published blood–brain barrier assembloid model, which is based on the assembly of pluripotent stem cell-derived brain organoids and blood vessel organoids, would provide a good basis for such an approach [40]. In combination with in vitro perfusion methods, this could also overcome a limitation of our study, in which the leakiness of KO vessels was not investigated.

In conclusion, blood vessel organoids differentiated according to our high-throughput compatible protocol are a useful addition to the repertoire of iPSC-based cell culture methods for biomedical research in general and CCM research in particular. On a methodological level, their scalability facilitates large-scale screening approaches, and on a disease-specific level, they provide new insights into the complex CCM disease. Our results suggest that CCM research should focus not only on endothelial dysfunction, but also on the role of perivascular cells and tumor-like mechanisms to find a new CCM therapy.

## Electronic supplementary material

Below is the link to the electronic supplementary material.

Fig. S1 Generation and quality control of CRISPR/Cas9 edited AICS-0016 and AICS-0036 *CCM1*, *CCM2* and *CCM3* KO hiPSC lines. Shown are the CCM1 (A), CCM2 (C), and CCM3 (E) protein and gene structures and the locations of CRISPR/Cas9 cleavage sites (top). Protein structures were adapted from Swamy and Glading 2022 [92]. Red indicators represent examples of *CCM1*, *CCM2,* and *CCM3* loss-of-function variants close to the CRISPR/Cas9 cleavage sites which are listed in ClinVar as pathogenic [NM_194454.3(*CCM1*):c.780C > G (p.Tyr260*), c.812G > A (p.Trp271*), c.857G > A (p.Trp286*); NM_031443.4(*CCM2*):c.228dup, (p.Pro77Thrfs*9), c.295del (p.His99Thrfs*7), c.305dup (p.His104Thrfs*35); NM_007217.4(*CCM3*): c.103C > T (p.Arg35*), c.160G > T (p.Glu54*), c.131dup (p.Arg45*)]. Representative sequencing results after CRISPR/Cas9 genome editing and single cell cloning shows homozygous or compound-heterozygous frameshift or nonsense variants in *CCM1* KO (A), *CCM2* KO (C), or *CCM3* KO (E) hiPSCs (bottom). Generated *CCM1* KO (B), *CCM2* KO (D), and *CCM3* KO hiPSCs (F) express the pluripotency markers TRA-1-60, OCT4, SOX2, and SSEA4 (representative images, scale bar: 200 µm). Karyotyping of knockout clones confirmed a normal karyotype (46, XY).

Fig. S2 No proliferative advantages of *CCM1* KO, *CCM2* KO, and *CCM3* KO hiPSCs. Cells were seeded on day 0 with a cell density of 2,000 cells per 96-well and cultured for 6 days. Nuclei were stained with Hoechst 33342, imaged on an Operetta CLS High-Content Analysis System and counted automatically with the Harmony High-Content Imaging and Analysis Software. Data are presented as mean ± SD of three independent experiments (n = 12 per condition). Normality was tested with the Shapiro–Wilk test. Statistical comparisons between groups were performed using multiple two-sample t-tests with Welch's correction to correct for unequal variances. The Holm-Šídák adjustment was applied within each outcome category to control for multiple comparisons. ns = not significant (P ≥ 0.05).

Fig. S3 *CCM1* KO, *CCM2* KO, and *CCM3* KO iECs show regular expression of endothelial markers and reorganization of the actin cytoskeleton compared to wild-type controls. **A** Immunofluorescence analysis of the endothelial markers CD31, VE-cadherin, and VWF in WT and KO AICS-0016-derived iECs (scale bar = 75 µm). **B** Endogenously tagged actin in AICS-0016 lines reveals actin stress fiber formation in *CCM* KO conditions (scale bar = 25 µm).
Supplementary Material 3

Fig. S4 Cell-type specificity analyses for scRNA-seq cell clusters. Results from WebCSEA showing the top 20 enriched general cell types for each cluster, which is summarized in the upper left panel. The results are based on the protein-coding marker genes with a normalized log_2_FC ≥ 0.5 for each cluster.
Supplementary Material 4

Fig. S5 Gene expression differences in the *CCM3* signature cluster 9.** A** The CZ CELLxGENE Discover browser was used to visualize the tissue and cell type-specific expression levels of the top 10 marker genes identified in cluster 9. Shown are cell types that are typically found in the brain and the vasculature. Purple color indicates high expression. Low expression is indicated by yellow color. The percentage of cells of the specific cell type that express the marker gene is visualized by the size of the circles. **B,C** Overlapping DEGs (B) in cluster 9 were subjected to a gene set enrichment analysis with the GO biological process gene set (C). **D,E** Heatmaps of gene expression differences for significantly up- (D) and downregulated (E) genes found in *CCM3* KO, but not *CCM1* KO or *CCM2* KO samples (= *CCM3* specific DEGs). Shown are the normalized average logFC values (KO vs. WT) for the three genotypes. ×  = Genes without expression information in *CCM1* and *CCM2* KO samples. **F,G***CCM3*-specific DEGs were subjected to gene set enrichment analyses with the GO cellular components (F) and molecular function (G) gene sets. Significantly up- and downregulated genes were defined as those with a normalized.avg_logFC (KO vs. WT) > 0.5 and p_adj_ < 0.05 or with a normalized.avg_logFC (KO vs. WT) < -0.5 and p_adj_ < 0.05, respectively.
Supplementary Material 5

Fig. S6 Gene expression differences in the *CCM1* signature cluster 8.** A** The CZ CELLxGENE Discover browser was used to visualize the tissue and cell type-specific expression levels of the top 10 marker genes identified in cluster 8. Shown are cell types that are typically found in the brain and the vasculature. Purple color indicates high expression. Low expression is indicated by yellow color. The percentage of cells of the specific cell type that express the marker gene is visualized by the size of the circles. **B**-**D** Genotype-specific gene expression differences in cluster 8 are shown in volcano plots for *CCM1* (B), *CCM2* (C), and *CCM3* (D) KO samples.** E,F** The overlaps of upregulated (E) and downregulated (F) genes in *CCM1*, *CCM2*, and *CCM3* KO cells are shown as Venn diagrams. **G***CCM1*-specific DEGs were subjected to a gene set enrichment analysis with the GO biological process gene set. Significantly up- and downregulated genes were defined as those with a normalized.avg_logFC (KO vs. WT) > 0.5 and p_adj_ < 0.05 or with a normalized.avg_logFC (KO vs. WT) < -0.5 and p_adj_ < 0.05, respectively.
Supplementary Material 6

Fig. S7 Gene expression differences in the *CCM2* signature cluster 3.** A** The CZ CELLxGENE Discover browser was used to visualize the tissue and cell type-specific expression levels of the top 10 marker genes identified in cluster 3. Shown are cell types that are typically found in the brain and the vasculature. Purple color indicates high expression. Low expression is indicated by yellow color. The percentage of cells of the specific cell type that express the marker gene is visualized by the size of the circles. **B,C** The overlaps of upregulated (B) and downregulated (C) genes in *CCM1*, *CCM2*, and *CCM3* KO cells are shown as Venn diagrams. **F***CCM2*-specific DEGs were subjected to a gene set enrichment analysis with the GO biological process gene set. Significantly up- and downregulated genes were defined as those with a normalized.avg_logFC (KO vs. WT) > 0.5 and p_adj_ < 0.05 or with a normalized.avg_logFC (KO vs. WT) < -0.5 and p_adj_ < 0.05, respectively.
Supplementary Material 7

Fig. S8 Structural differences in mosaic KO/WT vascular networks. AICS-0054 WT hiPSCs (mTagRFPT) were mixed with AICS-0036 *CCM1* KO, *CCM2* KO*, CCM3* KO or control WT hiPSCs (mEGFP) in a 19:1 ratio and differentiated to mosaic vascular networks. **A** Co-staining for the EC marker CD31 (blue) and the pericyte marker PDGFR-ß (red) showed the presence of KO cells (green) in the EC and pericyte cell populations (scale bar = 50 µm). Staining also indicated more convoluted EC and pericyte networks in mosaic *CCM3* KO/WT vascular networks. **B** Staining for the EC-specific adhesion molecule VE-cadherin identified many KO cells without proper VE-cadherin expression (white arrow heads) in mosaic *CCM3* KO/WT vascular networks (scale bar = 50 µm).
Supplementary Material 8

Video S1–S4 3D reconstructions of mosaic vascular networks show that most green-labeled *CCM1* (S1), *CCM2* (S2)*,* and *CCM3* KO cells (S3) do not express VE-cadherin (red) compared to the green-labeled WT control cells (S4). Vascular networks were generated by mixing AICS-0054 WT (mTagRFPT) hiPSCs with AICS-0036 *CCM1*, *CCM2, CCM3* KO, or WT hiPSCs in a 19:1 ratio and performing vascular network differentiation. 3D reconstructions of the green-labeled AICS-0036 derived cells and VE-cadherin stainings (red) were created with FIJI v.1.54 (S1 = *CCM1* KO, S2 = *CCM2* KO, S3 = *CCM3* KO, S4 = WT control).
Supplementary file9 (ZIP 41324 kb)

Table S1 Known lesion marker genes with normalized average logFC in each cluster. Lesion marker genes were adapted from Dao et al. 2024 [40].
Supplementary Material 10

## Data Availability

All relevant data are published within the paper and the supplementary files. ScRNA sequencing data can be accessed through the Gene Expression Omnibus (GEO) database (record number: GSE276497).
