# Supplementary figures and images for: High-throughput differentiation of human blood vessel organoids reveals overlapping and distinct functions of the cerebral cavernous malformation proteins
Source: Angiogenesis. 2025 Jun 6;28(3):32. doi: 10.1007/s10456-025-09985-5 (PMC12143994; doi:10.1007/s10456-025-09985-5)

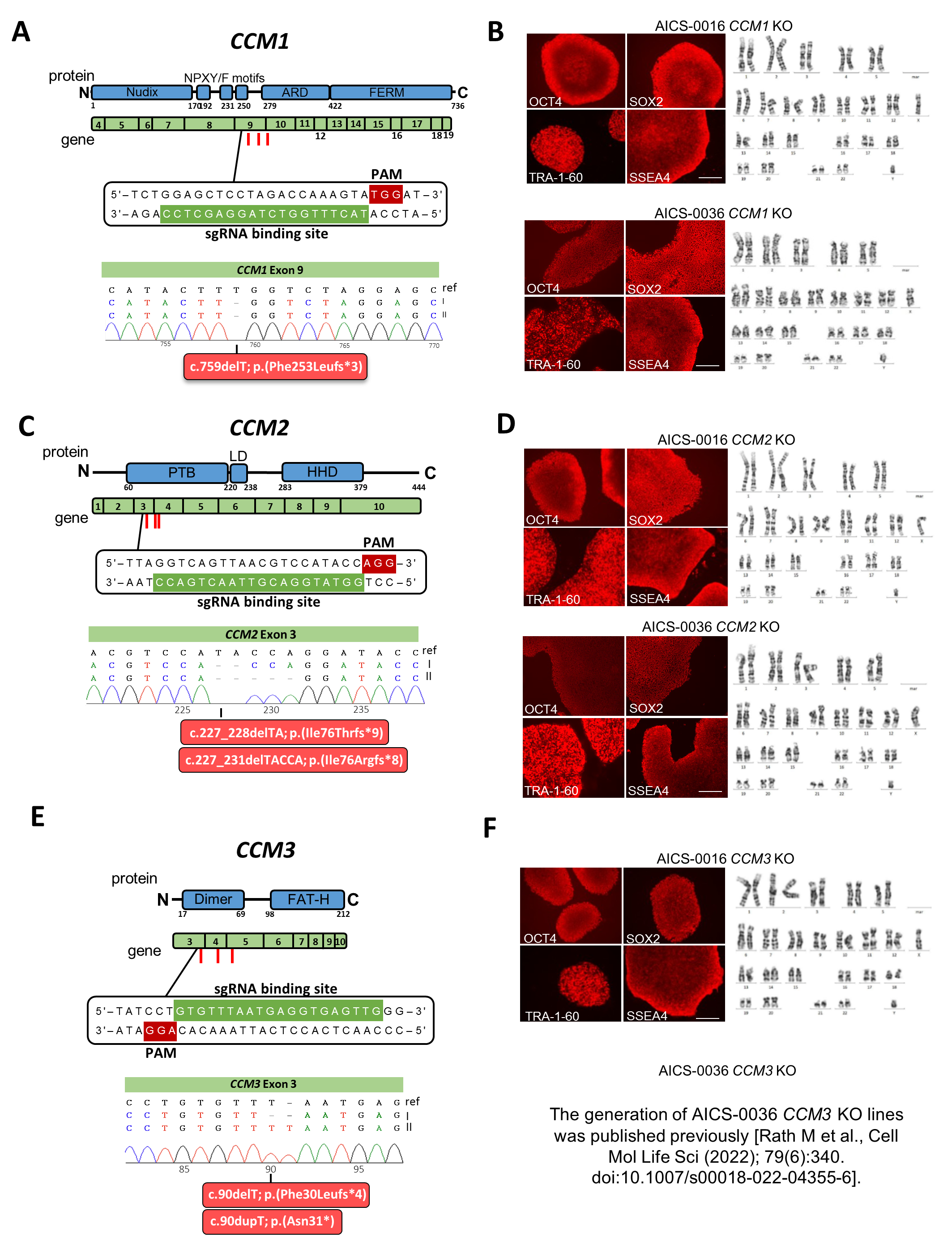

Supplement: Supplementary file 1 — Generation and quality control of CRISPR/Cas9 edited AICS-0016 and AICS-0036 CCM1, CCM2 and CCM3 KO hiPSC lines. Shown are the CCM1 (A), CCM2 (C), and CCM3 (E) protein and gene structures and the locations of CRISPR/Cas9 cleavage sites (top). Protein structures were adapted from Swamy and Glading 2022 [92]. Red indicators represent examples of CCM1, CCM2, and CCM3 loss-of-function variants close to the CRISPR/Cas9 cleavage sites which are listed in ClinVar as pathogenic [NM_194454.3(CCM1):c.780C > G (p.Tyr260*), c.812G > A (p.Trp271*), c.857G > A (p.Trp286*); NM_031443.4(CCM2):c.228dup, (p.Pro77Thrfs*9), c.295del (p.His99Thrfs*7), c.305dup (p.His104Thrfs*35); NM_007217.4(CCM3): c.103C > T (p.Arg35*), c.160G > T (p.Glu54*), c.131dup (p.Arg45*)]. Representative sequencing results after CRISPR/Cas9 genome editing and single cell cloning shows homozygous or compound-heterozygous frameshift or nonsense variants in CCM1 KO (A), CCM2 KO (C), or CCM3 KO (E) hiPSCs (bottom). Generated CCM1 KO (B), CCM2 KO (D), and CCM3 KO hiPSCs (F) express the pluripotency markers TRA-1-60, OCT4, SOX2, and SSEA4 (representative images, scale bar: 200 µm). Karyotyping of knockout clones confirmed a normal karyotype (46, XY). [file 10456_2025_9985_MOESM1_ESM.tif]

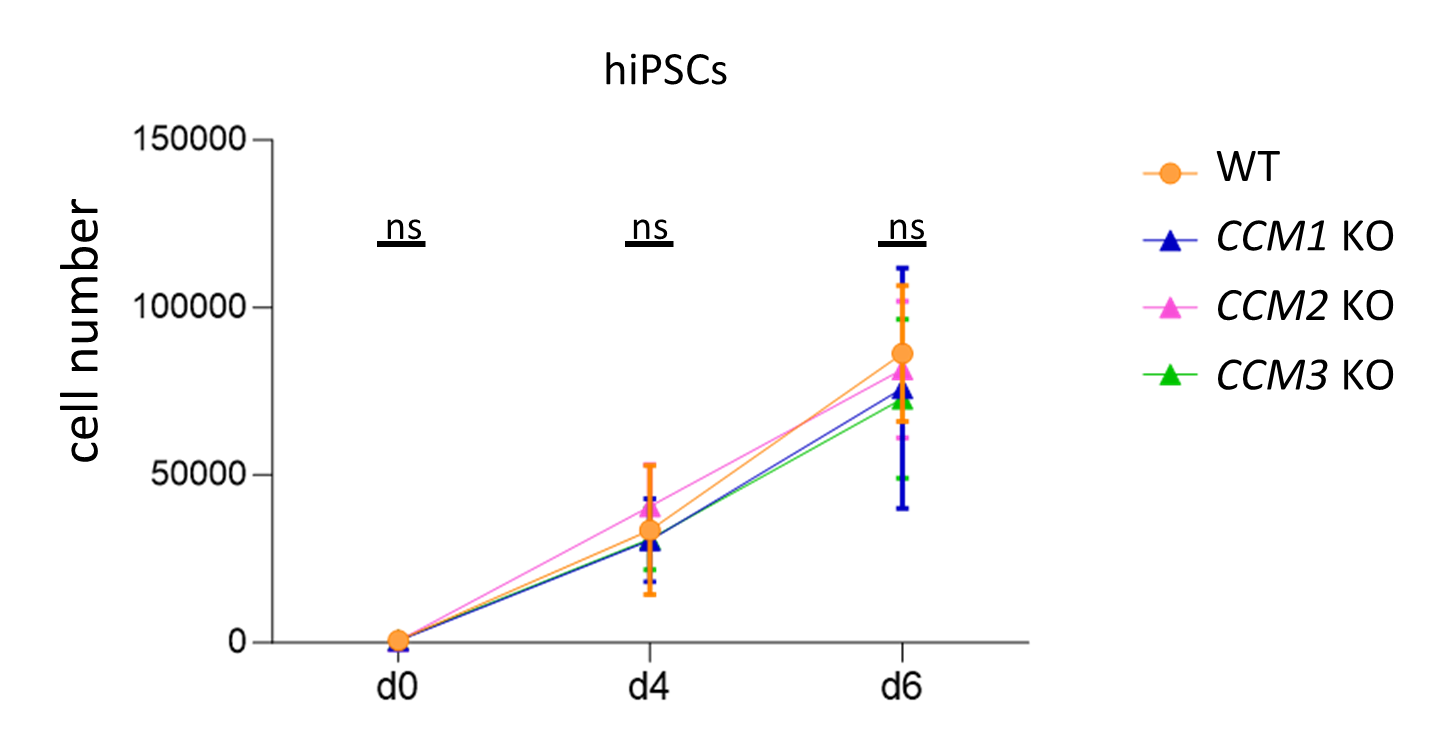

Supplement: Supplementary file 2 — No proliferative advantages of CCM1 KO, CCM2 KO, and CCM3 KO hiPSCs. Cells were seeded on day 0 with a cell density of 2,000 cells per 96-well and cultured for 6 days. Nuclei were stained with Hoechst 33342, imaged on an Operetta CLS High-Content Analysis System and counted automatically with the Harmony High-Content Imaging and Analysis Software. Data are presented as mean ± SD of three independent experiments (n = 12 per condition). Normality was tested with the Shapiro–Wilk test. Statistical comparisons between groups were performed using multiple two-sample t-tests with Welch's correction to correct for unequal variances. The Holm-Šídák adjustment was applied within each outcome category to control for multiple comparisons. ns = not significant (P ≥ 0.05). [file 10456_2025_9985_MOESM2_ESM.tif]

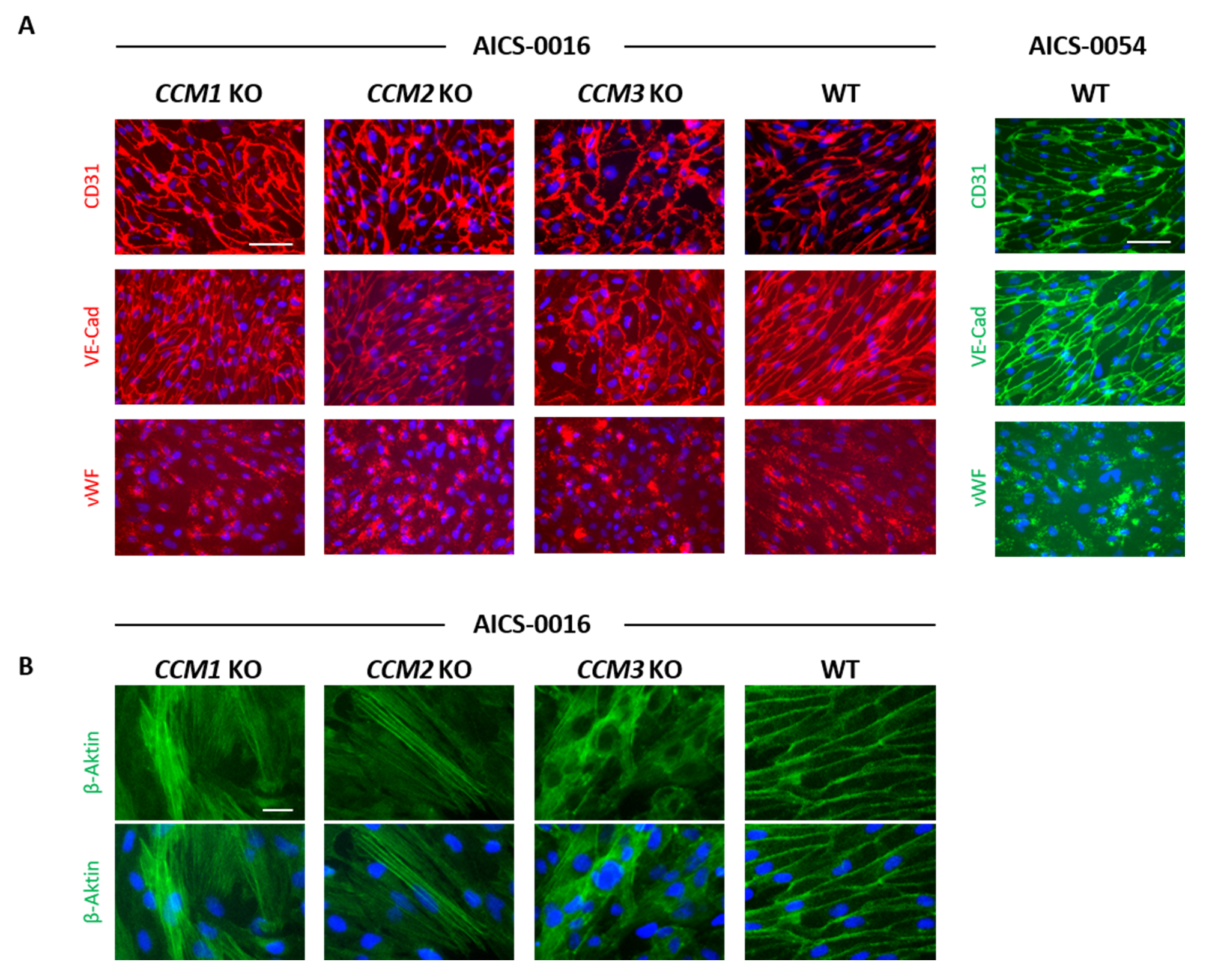

Supplement: Supplementary file 3 — CCM1 KO, CCM2 KO, and CCM3 KO iECs show regular expression of endothelial markers and reorganization of the actin cytoskeleton compared to wild-type controls. A Immunofluorescence analysis of the endothelial markers CD31, VE-cadherin, and VWF in WT and KO AICS-0016-derived iECs (scale bar = 75 µm). B Endogenously tagged actin in AICS-0016 lines reveals actin stress fiber formation in CCM KO conditions (scale bar = 25 µm). Supplementary Material 3 [file 10456_2025_9985_MOESM3_ESM.tif]

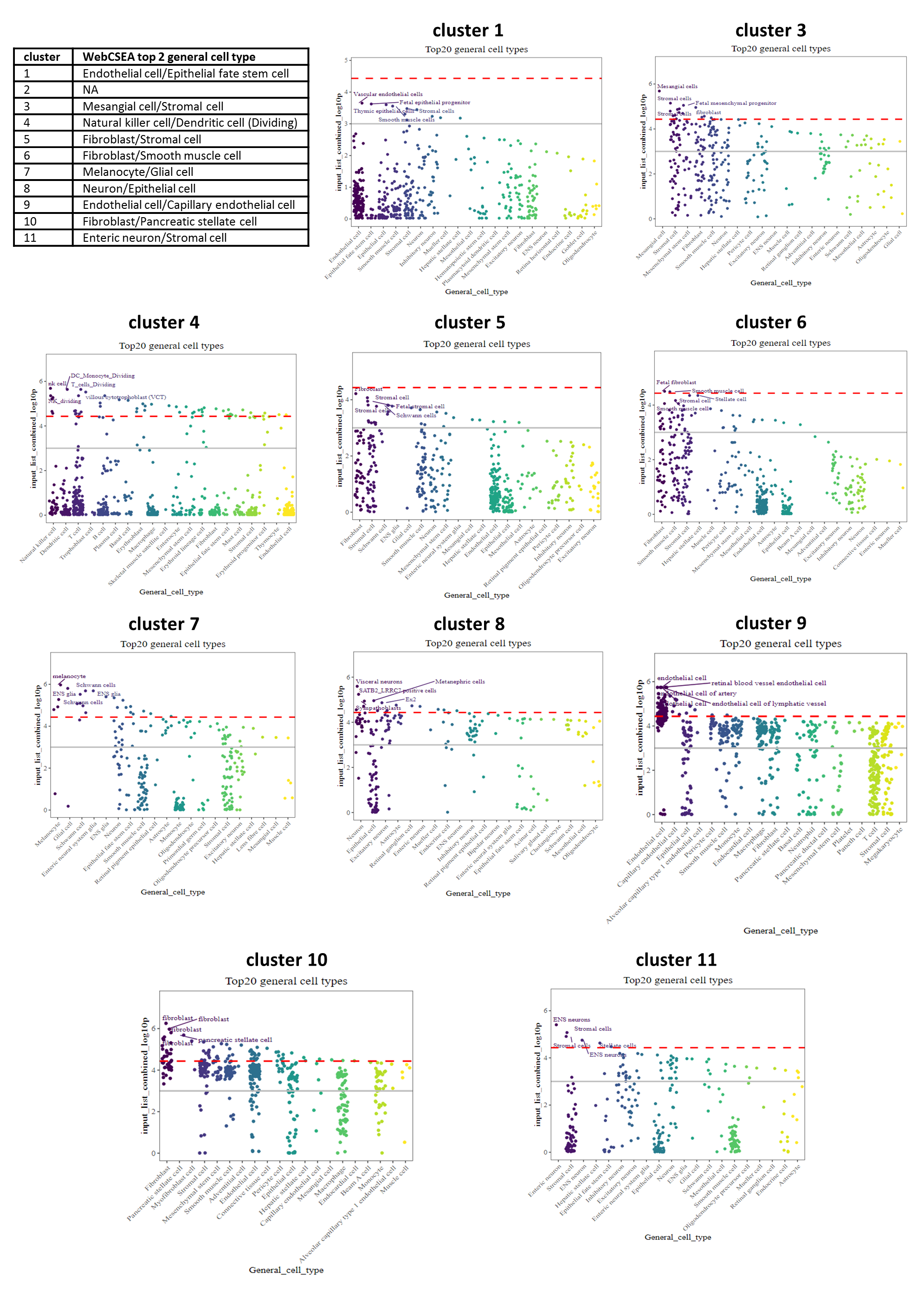

Supplement: Supplementary file 4 — Cell-type specificity analyses for scRNA-seq cell clusters. Results from WebCSEA showing the top 20 enriched general cell types for each cluster, which is summarized in the upper left panel. The results are based on the protein-coding marker genes with a normalized log2FC ≥ 0.5 for each cluster. Supplementary Material 4 [file 10456_2025_9985_MOESM4_ESM.tif]

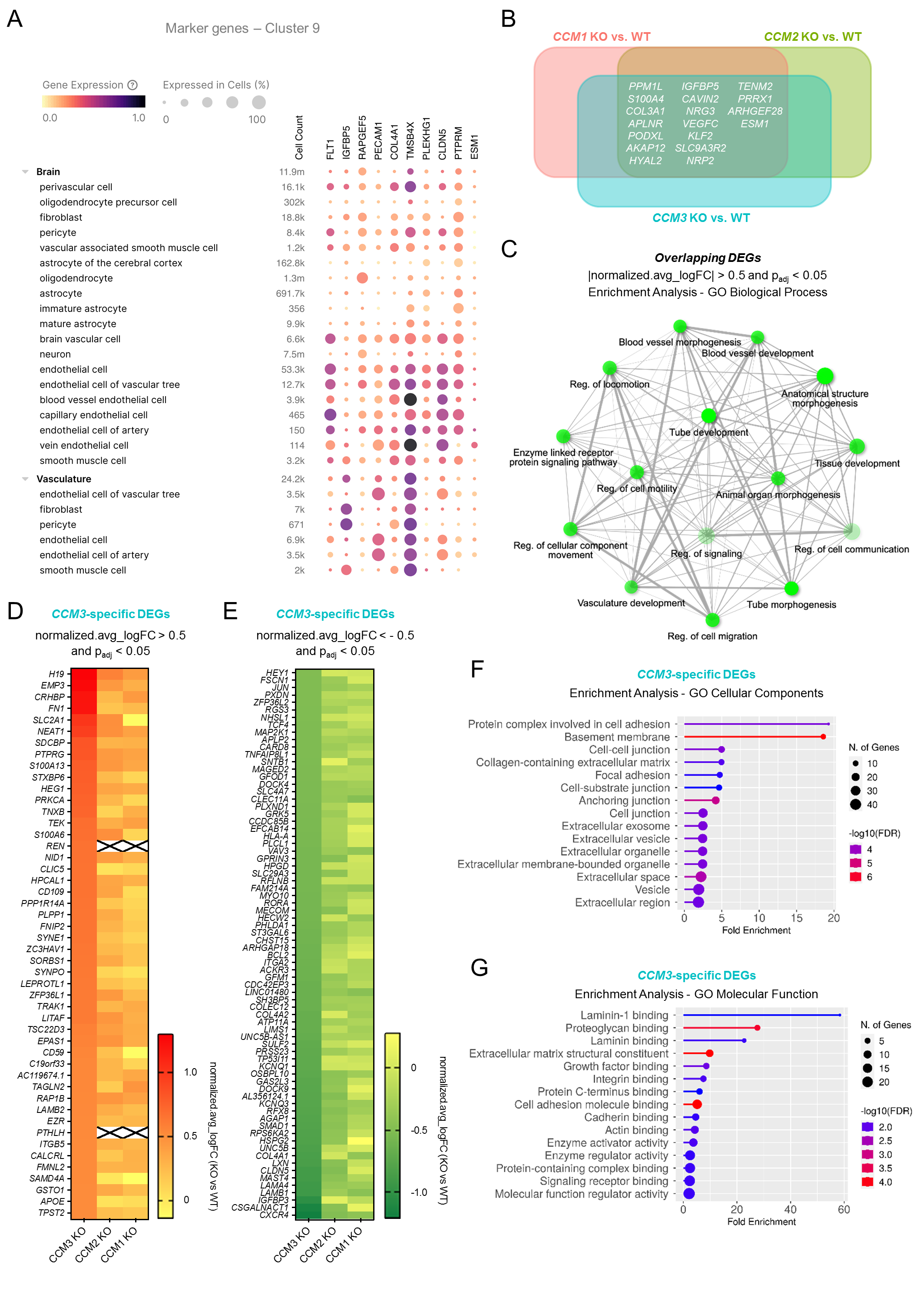

Supplement: Supplementary file 5 — Gene expression differences in the CCM3 signature cluster 9. A The CZ CELLxGENE Discover browser was used to visualize the tissue and cell type-specific expression levels of the top 10 marker genes identified in cluster 9. Shown are cell types that are typically found in the brain and the vasculature. Purple color indicates high expression. Low expression is indicated by yellow color. The percentage of cells of the specific cell type that express the marker gene is visualized by the size of the circles. B,C Overlapping DEGs (B) in cluster 9 were subjected to a gene set enrichment analysis with the GO biological process gene set (C). D,E Heatmaps of gene expression differences for significantly up- (D) and downregulated (E) genes found in CCM3 KO, but not CCM1 KO or CCM2 KO samples (= CCM3 specific DEGs). Shown are the normalized average logFC values (KO vs. WT) for the three genotypes. × = Genes without expression information in CCM1 and CCM2 KO samples. F,GCCM3-specific DEGs were subjected to gene set enrichment analyses with the GO cellular components (F) and molecular function (G) gene sets. Significantly up- and downregulated genes were defined as those with a normalized.avg_logFC (KO vs. WT) > 0.5 and padj < 0.05 or with a normalized.avg_logFC (KO vs. WT) < -0.5 and padj < 0.05, respectively. Supplementary Material 5 [file 10456_2025_9985_MOESM5_ESM.tif]

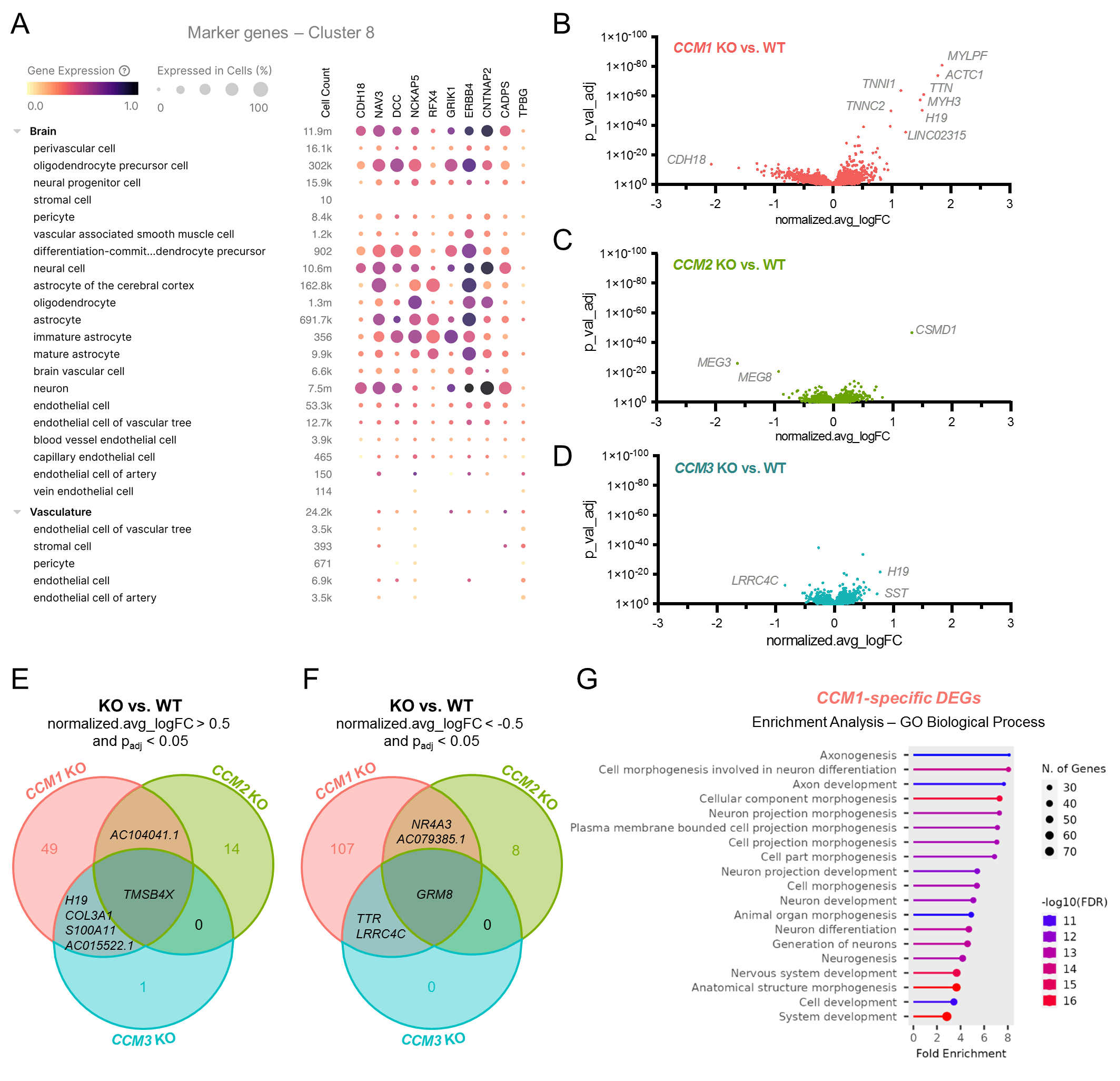

Supplement: Supplementary file 6 — Gene expression differences in the CCM1 signature cluster 8. A The CZ CELLxGENE Discover browser was used to visualize the tissue and cell type-specific expression levels of the top 10 marker genes identified in cluster 8. Shown are cell types that are typically found in the brain and the vasculature. Purple color indicates high expression. Low expression is indicated by yellow color. The percentage of cells of the specific cell type that express the marker gene is visualized by the size of the circles. B-D Genotype-specific gene expression differences in cluster 8 are shown in volcano plots for CCM1 (B), CCM2 (C), and CCM3 (D) KO samples. E,F The overlaps of upregulated (E) and downregulated (F) genes in CCM1, CCM2, and CCM3 KO cells are shown as Venn diagrams. GCCM1-specific DEGs were subjected to a gene set enrichment analysis with the GO biological process gene set. Significantly up- and downregulated genes were defined as those with a normalized.avg_logFC (KO vs. WT) > 0.5 and padj < 0.05 or with a normalized.avg_logFC (KO vs. WT) < -0.5 and padj < 0.05, respectively. Supplementary Material 6 [file 10456_2025_9985_MOESM6_ESM.tif]

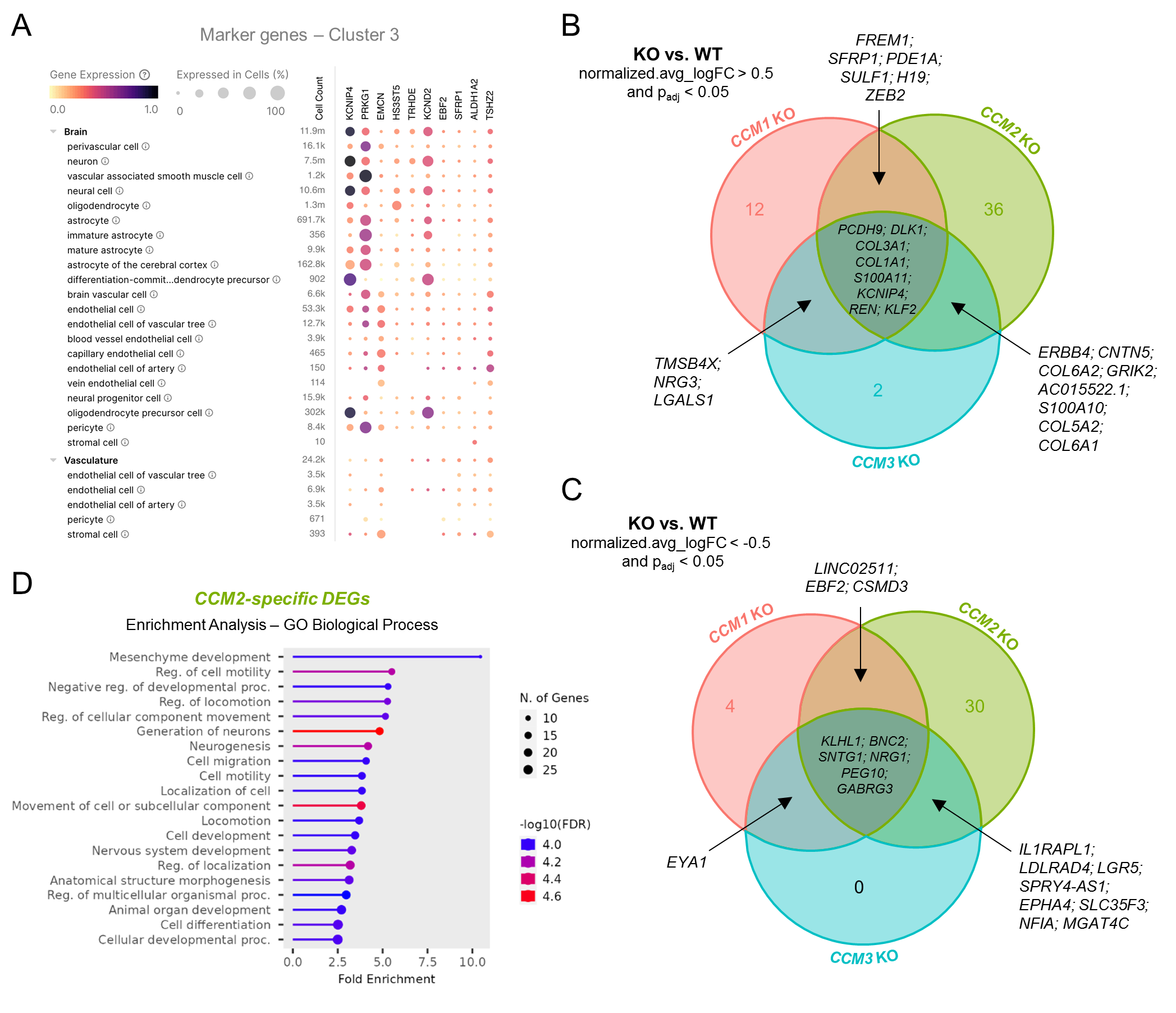

Supplement: Supplementary file 7 — Gene expression differences in the CCM2 signature cluster 3. A The CZ CELLxGENE Discover browser was used to visualize the tissue and cell type-specific expression levels of the top 10 marker genes identified in cluster 3. Shown are cell types that are typically found in the brain and the vasculature. Purple color indicates high expression. Low expression is indicated by yellow color. The percentage of cells of the specific cell type that express the marker gene is visualized by the size of the circles. B,C The overlaps of upregulated (B) and downregulated (C) genes in CCM1, CCM2, and CCM3 KO cells are shown as Venn diagrams. FCCM2-specific DEGs were subjected to a gene set enrichment analysis with the GO biological process gene set. Significantly up- and downregulated genes were defined as those with a normalized.avg_logFC (KO vs. WT) > 0.5 and padj < 0.05 or with a normalized.avg_logFC (KO vs. WT) < -0.5 and padj < 0.05, respectively. Supplementary Material 7 [file 10456_2025_9985_MOESM7_ESM.tif]

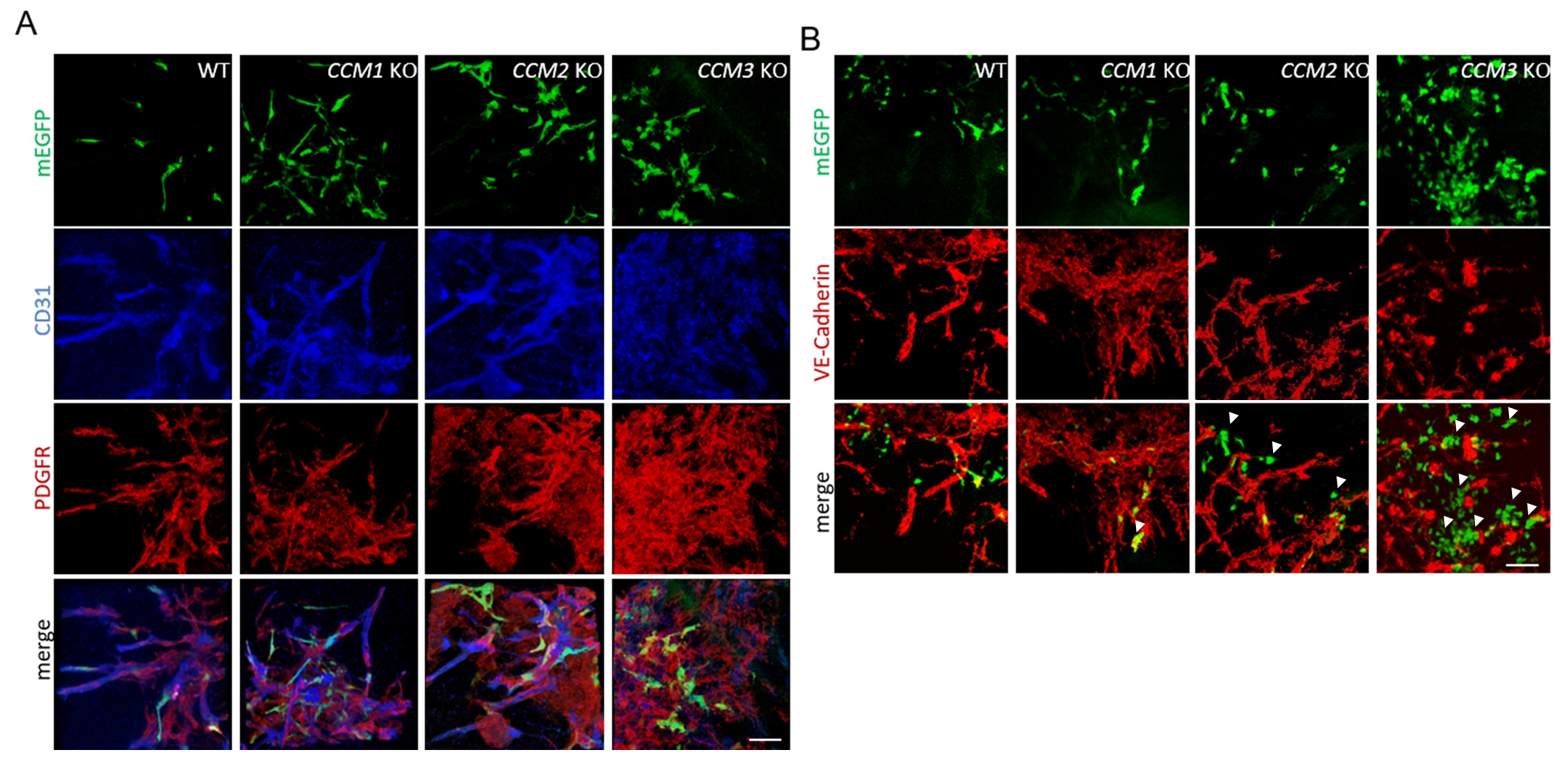

Supplement: Supplementary file 8 — Structural differences in mosaic KO/WT vascular networks. AICS-0054 WT hiPSCs (mTagRFPT) were mixed with AICS-0036 CCM1 KO, CCM2 KO, CCM3 KO or control WT hiPSCs (mEGFP) in a 19:1 ratio and differentiated to mosaic vascular networks. A Co-staining for the EC marker CD31 (blue) and the pericyte marker PDGFR-ß (red) showed the presence of KO cells (green) in the EC and pericyte cell populations (scale bar = 50 µm). Staining also indicated more convoluted EC and pericyte networks in mosaic CCM3 KO/WT vascular networks. B Staining for the EC-specific adhesion molecule VE-cadherin identified many KO cells without proper VE-cadherin expression (white arrow heads) in mosaic CCM3 KO/WT vascular networks (scale bar = 50 µm). Supplementary Material 8 [file 10456_2025_9985_MOESM8_ESM.tif]

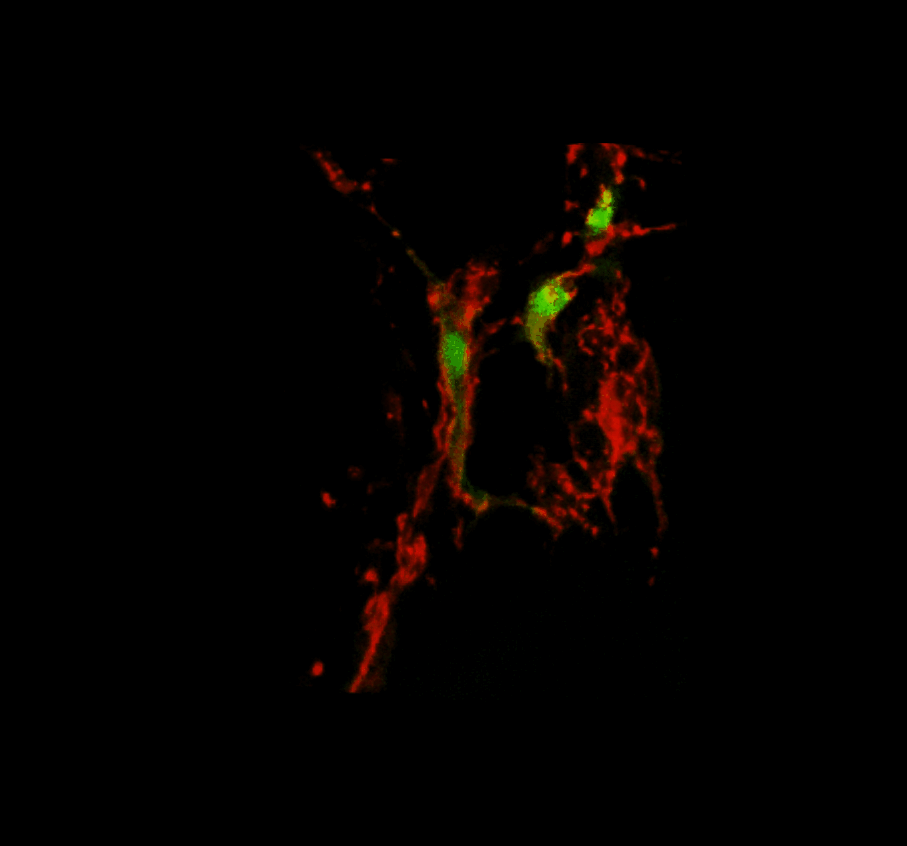

Supplement: Supplementary file 9 — 3D reconstructions of mosaic vascular networks show that most green-labeled CCM1 (S1), CCM2 (S2), and CCM3 KO cells (S3) do not express VE-cadherin (red) compared to the green-labeled WT control cells (S4). Vascular networks were generated by mixing AICS-0054 WT (mTagRFPT) hiPSCs with AICS-0036 CCM1, CCM2, CCM3 KO, or WT hiPSCs in a 19:1 ratio and performing vascular network differentiation. 3D reconstructions of the green-labeled AICS-0036 derived cells and VE-cadherin stainings (red) were created with FIJI v.1.54 (S1 = CCM1 KO, S2 = CCM2 KO, S3 = CCM3 KO, S4 = WT control). Supplementary file9 (ZIP 41324 kb) [file 10456_2025_9985_MOESM9_ESM.zip › 10456_2025_9985_MOESM9_ESM/Sup_Video_S1 (CCM1).gif]

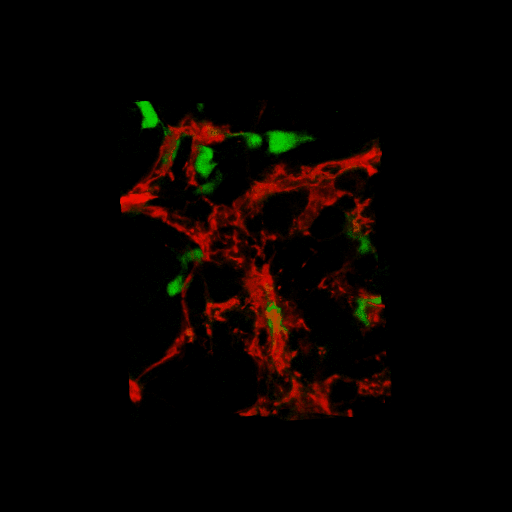

Supplement: Supplementary file 9 — 3D reconstructions of mosaic vascular networks show that most green-labeled CCM1 (S1), CCM2 (S2), and CCM3 KO cells (S3) do not express VE-cadherin (red) compared to the green-labeled WT control cells (S4). Vascular networks were generated by mixing AICS-0054 WT (mTagRFPT) hiPSCs with AICS-0036 CCM1, CCM2, CCM3 KO, or WT hiPSCs in a 19:1 ratio and performing vascular network differentiation. 3D reconstructions of the green-labeled AICS-0036 derived cells and VE-cadherin stainings (red) were created with FIJI v.1.54 (S1 = CCM1 KO, S2 = CCM2 KO, S3 = CCM3 KO, S4 = WT control). Supplementary file9 (ZIP 41324 kb) [file 10456_2025_9985_MOESM9_ESM.zip › 10456_2025_9985_MOESM9_ESM/Sup_Video_S2 (CCM2).gif]

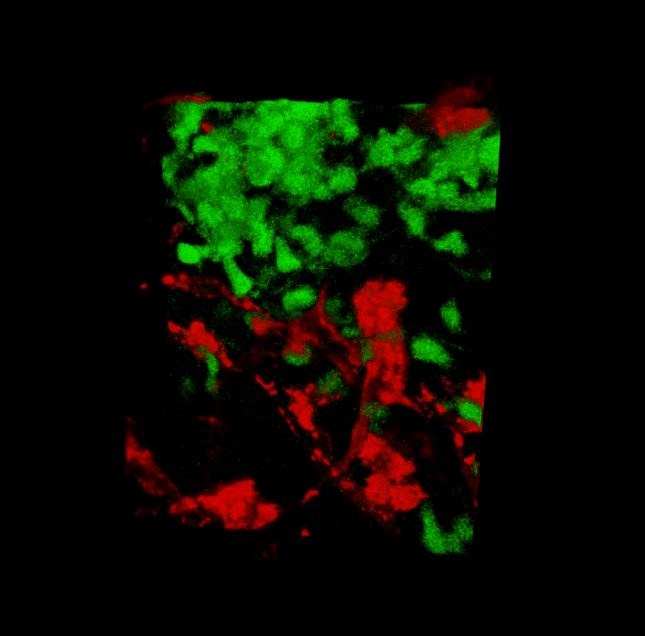

Supplement: Supplementary file 9 — 3D reconstructions of mosaic vascular networks show that most green-labeled CCM1 (S1), CCM2 (S2), and CCM3 KO cells (S3) do not express VE-cadherin (red) compared to the green-labeled WT control cells (S4). Vascular networks were generated by mixing AICS-0054 WT (mTagRFPT) hiPSCs with AICS-0036 CCM1, CCM2, CCM3 KO, or WT hiPSCs in a 19:1 ratio and performing vascular network differentiation. 3D reconstructions of the green-labeled AICS-0036 derived cells and VE-cadherin stainings (red) were created with FIJI v.1.54 (S1 = CCM1 KO, S2 = CCM2 KO, S3 = CCM3 KO, S4 = WT control). Supplementary file9 (ZIP 41324 kb) [file 10456_2025_9985_MOESM9_ESM.zip › 10456_2025_9985_MOESM9_ESM/Sup_Video_S3 (CCM3).gif]

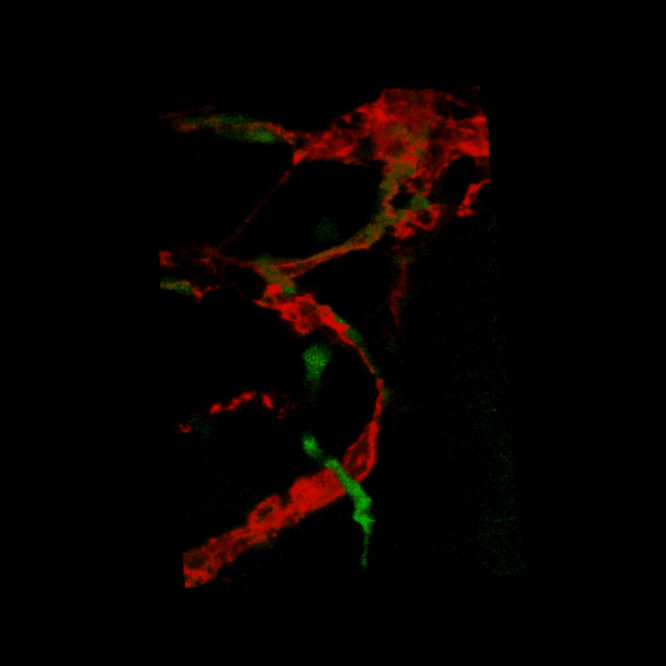

Supplement: Supplementary file 9 — 3D reconstructions of mosaic vascular networks show that most green-labeled CCM1 (S1), CCM2 (S2), and CCM3 KO cells (S3) do not express VE-cadherin (red) compared to the green-labeled WT control cells (S4). Vascular networks were generated by mixing AICS-0054 WT (mTagRFPT) hiPSCs with AICS-0036 CCM1, CCM2, CCM3 KO, or WT hiPSCs in a 19:1 ratio and performing vascular network differentiation. 3D reconstructions of the green-labeled AICS-0036 derived cells and VE-cadherin stainings (red) were created with FIJI v.1.54 (S1 = CCM1 KO, S2 = CCM2 KO, S3 = CCM3 KO, S4 = WT control). Supplementary file9 (ZIP 41324 kb) [file 10456_2025_9985_MOESM9_ESM.zip › 10456_2025_9985_MOESM9_ESM/Sup_Video_S4 (WT).gif]
